# ADAM15 mediates upregulation of Claudin-1 expression in breast cancer cells

**DOI:** 10.1038/s41598-019-49021-3

**Published:** 2019-08-29

**Authors:** Jens Mattern, Christian S. Roghi, Melanie Hurtz, Vera Knäuper, Dylan R. Edwards, Zaruhi Poghosyan

**Affiliations:** 1 Division of Cancer and Genetics, School of Medicine, College of Biomedical and Life Sciences, Cardiff University Heath Park, Cardiff, CF14 4XN UK; 2 Oral and Biomedical Sciences, School of Dentistry, College of Biomedical and Life Sciences, Cardiff University, Heath Park, Cardiff, CF14 4XY UK; 3 School of Biological Sciences and Norwich Medical School, University of East Anglia, Norwich Research Park, Norwich, NR4 7TJ UK; 4 Present Address: Quadram Institute Bioscience, Norwich Research Park, Norwich, NR4 7UA UK; 5 Present Address: MLM Medical Labs GmbH, Dohrweg 63, 41066 Mönchengladbach, Germany

**Keywords:** Breast cancer, Mechanisms of disease

## Abstract

A Disintegrin and Metalloproteinase-15 (ADAM15) is a transmembrane protein involved in protein ectodomain shedding, cell adhesion and signalling. We previously cloned and characterised alternatively spliced variants of ADAM15 that differ in their intracellular domains and demonstrated correlation of the expression of specific variants with breast cancer prognosis. In this study we have created isogenic cell panels (MDA-MB-231 and MCF-7) expressing five ADAM15 variants including wild-type and catalytically inactive forms. The expression of ADAM15 isoforms in MDA-MB-231 cells led to cell clustering to varying degree, without changes in EMT markers vimentin, slug and E-cadherin. Analysis of tight junction molecules revealed ADAM15 isoform specific, catalytic function dependent upregulation of Claudin-1. The expression of ADAM15A, and to a lesser degree of C and E isoforms led to an increase in Claudin-1 expression in MDA-MB-231 cells, while ADAM15B had no effect. In MCF-7 cells, ADAM15E was the principal variant inducing Claudin-1 expression. Sh-RNA mediated down-regulation of ADAM15 in ADAM15 over-expressing cells reduced Claudin-1 levels. Additionally, downregulation of endogenous ADAM15 expression in T47D cells by shRNA reduced endogenous Claudin-1 expression confirming a role for ADAM15 in regulating Claudin-1 expression. The PI3K/Akt/mTOR pathway was involved in regulating Claudin-1 expression downstream of ADAM15. Immunofluorescence analysis of MDA-MB-231 ADAM15A expressing cells showed Claudin-1 at cell-cell junctions, in the cytoplasm and nuclei. ADAM15 co-localised with Claudin-1 and ZO1 at cell-cell junctions. Immunoprecipitation analysis demonstrated complex formation between ADAM15 and ZO1/ZO2. These findings highlight the importance of ADAM15 Intra Cellular Domain-mediated interactions in regulating substrate selection and breast cancer cell phenotype.

## Introduction

The ADAM (a metalloproteinase and a disintegrin) family of transmembrane and secreted metalloproteinases perform important functions in cell adhesion and signalling, primarily through the regulated ectodomain shedding of ligands and receptors for multiple pathways such as those controlled by the epidermal growth factor receptor and Notch^1,2^. ADAM15 shares the multi-domain organisation of other family members, containing metalloproteinase (MP), disintegrin (dis), cysteine-rich and EGF-like domains, which are connected via a transmembrane helix to the intracellular domain (ICD)^3^. The ADAM15 ICD contains tyrosines that when phosphorylated interact with SH2 domain containing proteins, and includes specific proline-rich regions that act as ligands for several SH3 domain containing proteins^4,5^. Additionally, we have shown that some of the interactions between ADAM15 and SH3 domain containing proteins depend on the phosphorylation status of the ADAM15 ICD^4^.

ADAM15 pre-mRNA is subject to complex alternative splicing in normal tissues and cancer cells leading to inclusion or exclusion of exons 18 to 23. As a result 13 isoforms of ADAM15 protein arise, that vary by the number and nature of their proline-rich regions, affecting ADAM15 ICD-mediated intracellular protein-protein interactions^5,6^. Previously we have shown that differential expression of ADAM15 spliced isoforms has prognostic significance in breast cancer patients^5^. In particular, the expression of ADAM15A/B isoforms correlate with poor prognosis in node-negative breast cancer patients, while the expression of ADAM15C correlates with better prognosis in node-positive disease^5^.

ADAM15 is catalytically active and has been shown to shed several transmembrane proteins such as CD23^7^, pro-amphiregulin and pro-HB-EGF^8^, E-cadherin^9^, MICB^10^, FGFR2IIIb^11^. Interestingly, we have shown that the shedding of FGFR2IIIb is ADAM15 isoform- and phosphorylation-dependent, with ADAM15B shedding efficiency enhanced through Src-dependent phosphorylation^12^.

To better understand how ADAM15 isoforms affect breast cancer prognosis, we have established two isogenic cell panels expressing individual ADAM15 isoforms in either the aggressive triple negative breast cancer cell line MDA-MB-231 or the less aggressive MCF7 cell line utilising the Invitrogen FLP-in system. This ensures that any changes observed in cell behaviour within the panel depend only on the ADAM15 isoform expressed and excludes gene copy number or integration artefacts. We show for the first time ADAM15 isoform-, and catalytic function-dependent upregulation of Claudin-1 expression, which may be responsible for the effects of ADAM15 on cancer cell phenotypes and prognostic outcomes for breast cancer patients.

## Materials and Methods

### Cell lines

The following cell lines and their derivatives were used in the study: Breast cancer cell lines MDA-MB-231, MCF7/FRT, MCF7/FRT, T47D, squamous cell carcinoma cell line A431, human embryonic kidney cell line HEK293FT. Cells were kept in culture up to three months after thawing. Cells were tested for mycoplasma by PCR analysis every 4–6 months. Selected cell lines were authenticated in 2014 by DNA Diagnostic Center (MDA-MB-231 and derivatives), and in 2018 by Eurofins (MCF7/FRT, T47D and derivatives).

### Generation of MDA-MB-231/FRT cells, and an isogenic ADAM15 expression panel

The Invitrogen Flp-In- system (Invitrogen K601001) was used to generate the isogenic cell panel expressing ADAM15 isoforms. Briefly, MDA-MB-231 cells were transfected with *ScaI* linearised pFRT/lacZeo plasmid using Lipofectamine 2000 (Invitrogen 11668019). Zeocin-resistant clones were isolated and screened for single integration sites by Southern Blot analysis, followed by low-coverage whole genome sequencing (BGI, Beijing). The validated MDA-MB-231 clone containing a single recombination site was co-transfected with pOG44, expressing Flp recombinase, and pcDNA5/FRT-V5-His plasmids encoding the ADAM15 WT and E349A isoforms. These plasmids were created by sub cloning the ADAM15 sequences from pcDNA4-V5/His ADAM15 expression plasmids^5^ using HindIII and XhoI into pcDNA5/FRT-V5-His vector. The cells were selected in DMEM containing 1 mg/ml Hygromycin B (Invitrogen).

### MTS proliferation assay

Cells were seeded in triplicates into four 96-well plates with 3 × 10^3^ cells per well in 100 µL volume. At indicated time points 20 µL of CellTiter 96 AQueous One Solution (Promega G3580) was added to each well and incubated for 4 h at 37 °C. The absorbance was measured at 450 nm. The measurement after 24 h was considered as 1.

### Demethylation and deacetylation

Cells were treated with 50 µM Decitabine (5-aza-2′-deoxycytidine) for three days, each day replacing the media and adding Decitabine fresh. On the third day, 500 nM Trichostatin A was added, and cells were lysed the following day.

### Flow cytometric analysis of cell size

Trypsinised cells were stained with 10 µg/ml propidium iodide and analysed by Accuri C6 flow cytometre. The median size was automatically calculated by FlowJo. For comparison, a scatter plot was generated and one-way ANOVA was performed.

### Microscopic determination of cell spread area

Phase-contrast images were taken at 200 x magnification and quantified with ImageJ. For each cell line five images of random positions were taken using an EVOS XL core imaging system (Thermo Fisher Scientific). This was done for three different passages to achieve 15 images. In each picture ten representative cells were chosen and the cell area was outlined and measured. This led to 150 measured cells for each cell line. For statistical analysis Bartlett’s test for equal variances was used to determine the significance. As follow-up test Dunnett’s multiple comparison test was used. The results are presented as a box-and-whiskers plot.

### Immunoprecipitation

Cells were lysed in RIPA buffer supplemented with proteinase and phosphatase inhibitors (Roche) and 10 mM phenanthroline (Sigma), and quantified using the BCA kit (Thermo Fisher). 500 µg/ml of total protein extract was used per IP. ADAM15 was IP-ed with α-V5 affinity gel (Sigma). For all other IPs Protein G sepharose was used together with respective antibodies. SDS-PAGE and Western Blotting were carried out using standard protocols.

### Scratch-wound assay

Cells were seeded into 6 cm dishes and grown until confluent. Wounds were introduced with a white tip (10 µL). Pictures were taken at 0, then at 8, 24, 32 and 48 hrs using an EVOS XL core cell imaging system (Thermo Fisher Scientific). Analysis was carried out with the MRI wound healing tool for ImageJ. The 0 h time points were normalised to 100% and the wound closure was calculated using Microsoft Excel. Statistical analysis was carried out with 2-way ANOVA and Bonferroni post-test in GraphPad Prism 5.01. ADAM15 isoform expressing cells were compared to the parental cell line MDA-MB-231/FRT.

### Immunostaining and confocal microscopy

Cells were seeded onto glass cover slips preincubated in complete growth media and grown for 72 hrs. Cells were washed with PBS, fixed 4% formaldehyde for 20 min, washed in PBS, then permeabilised with 0.1% saponin/PBS for 2 min. After further washes with PBS, cells were blocked for 30 min in 1% BSA in PBS. Afterwards, the cells were incubated with the primary antibodies overnight at 4 °C in a humidified chamber. After further washes with PBS, corresponding Alexa conjugates were added in the blocking buffer for 1 hr. Excess antibodies were washed off with PBS, and the cover slips were mounted on microscope slides using Prolong Gold mounting media with DAPI (Invitrogen). Slides were analysed using a Leica SP5 confocal microscope (Leica, Microsystems) and images acquired with a 63x oil immersion objective.

### Lentiviral particle production and shRNA-mediated downregulation

pLKO.1/puromycin plasmids encoding Mission shRNA (Sigma) sequences were used (table 4 in Supplementary Information). Additionally, an shRNA sequence targeting the 3′ UTR of ADAM15: FWD 5′-CCGGGTTGGACGGGATTGAGGAACTCGAGTTCCTCAATCCCGTCCAACTTTTTG-3′ REV 5′-AATTCAAAAAGTTGGACGGGATTGAGGAACTCGAGTTCCTCAATCCCGTCCAAC-3′, was cloned into pLKO.1 using AgeI and EcorRI restriction sites. The positive clones were selected by XhoI digest according to Sigma Mission shRNA protocol. Lentiviral particles were produced according to Sigma Mission shRNA protocol. Stable downregulation of Claudin-1 or ADAM15 was achieved by 1 µg/mL puromycin selection.

### RNA extraction, RT-PCR and qPCR

Qiagen QIAshredder (Qiagen 79654), RNeasy Kit (Qiagen 74104) and Superscript II (Invitrogen 18064014) were used to extract RNA and perform RT. KAPA 2G Robust Hot Start ready PCR mix (KAPA Biosystems KK5702) was used to amplify Claudin-1 and GAPDH (Table 5 in Supplementary information). qPCR by Taqman analysis for claudin-1 expression was performed using Hs00221623_M1 primer and probe set (Thermo Fisher Scientific).

## Results

### Generation of an isogenic panel expressing proteolytically active and inactive ADAM15 isoforms in MDA-MB-231 cells

**Figure 1:**
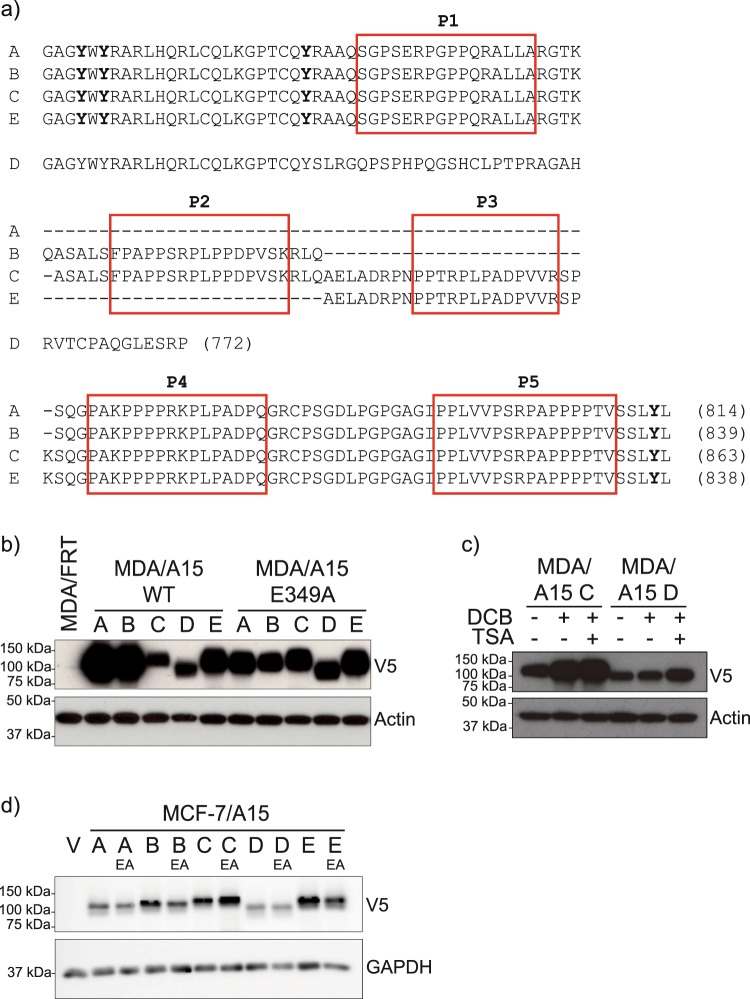
Isogenic cell panels expressing individual ADAM15 wildtype and catalytically inactive isoforms in MDA-MB-231 and MCF-7 cells. (**a**) Schematic representation of ADAM15 ICD variants. Proline-rich regions are boxed. (**b**) Expression of ADAM15 isoforms in MDA-MB-231 cells, (**c**) MDA/ADAM15 C and D cells treated with 50 µM Decitabine for 72hrs, each 24 hr replacing the media and adding Decitabine fresh. For the last 24hrs, 500 nM Trichostatin A was added, followed by analysis for ADAM15 expression. (**d**) Expression level of ADAM15 isoforms in MCF-7 cells.

Alternatively-spliced ADAM15 isoforms (Fig. 1a) have prognostic significance in breast cancer, implying that the isoforms affect cell behaviour differently^5^. To elucidate the role of ADAM15 isoforms in breast cancer progression, we generated an isogenic cell panel expressing each ADAM15 isoform in MDA-MB-231 cells. To achieve that, we introduced an FRT site for the FLP recombinase into MDA-MB-231 cells, and selected a clone containing a single integration site. The selected MDA-MB-231/FRT clone was confirmed by next-generation sequencing to contain a single integration site on chromosome 4 within the intron separating exons 5 and 6 of the *FAM190A* gene. Analysis of the endogenous expression levels of individual ADAM15 isoforms in this clone by qPCR demonstrated the presence of predominantly ADAM15A isoform (Supplementary Fig. 1a).

We used this MDA-MB-231/FRT clone to co-transfect with the pOG44 plasmid encoding the FLP recombinase together with pcDNA5/FRT plasmids encoding the five main ADAM15 isoforms in both WT and catalytically-dead E349A versions, each of which carried a C-terminal V5-His tag for detection (Fig. 1b). The presence of the ADAM15 cassettes had no effect on expression of the host *FAM190A* gene (Supplementary Figure 1b).

In the isogenic panel of MDA-MB-231/FRT cell lines we noted some variation in the levels of expression of the isoforms, with slightly higher expression of WT ADAM15A and ADAM15B, and lower levels of WT ADAM15C and ADAM15D isoforms (Fig. 1b). We asked if differential methylation is responsible for varying expression levels of ADAM15 isoforms. Treatment of cells with DNA demethylating agent Decitabine led to an increase in the expression levels of ADAM15, which was further enhanced by a combined treatment with HDAC inhibitor trichostatinA, validating that the decreased expression of some isoforms is due to epigenetic suppression (Fig. 1c). For comparison, we also generated an isogenic cell panel expressing ADAM15 isoforms in MCF7/FRT cells^13^. The expression level of ADAM15 isoforms within the MCF7 panel was nearly equal in this series, though again ADAM15D showed the lowest steady-state level (Fig. 1d).

### Expression of ADAM15 isoforms affects cell morphology, proliferation and migration

**Figure 2:**
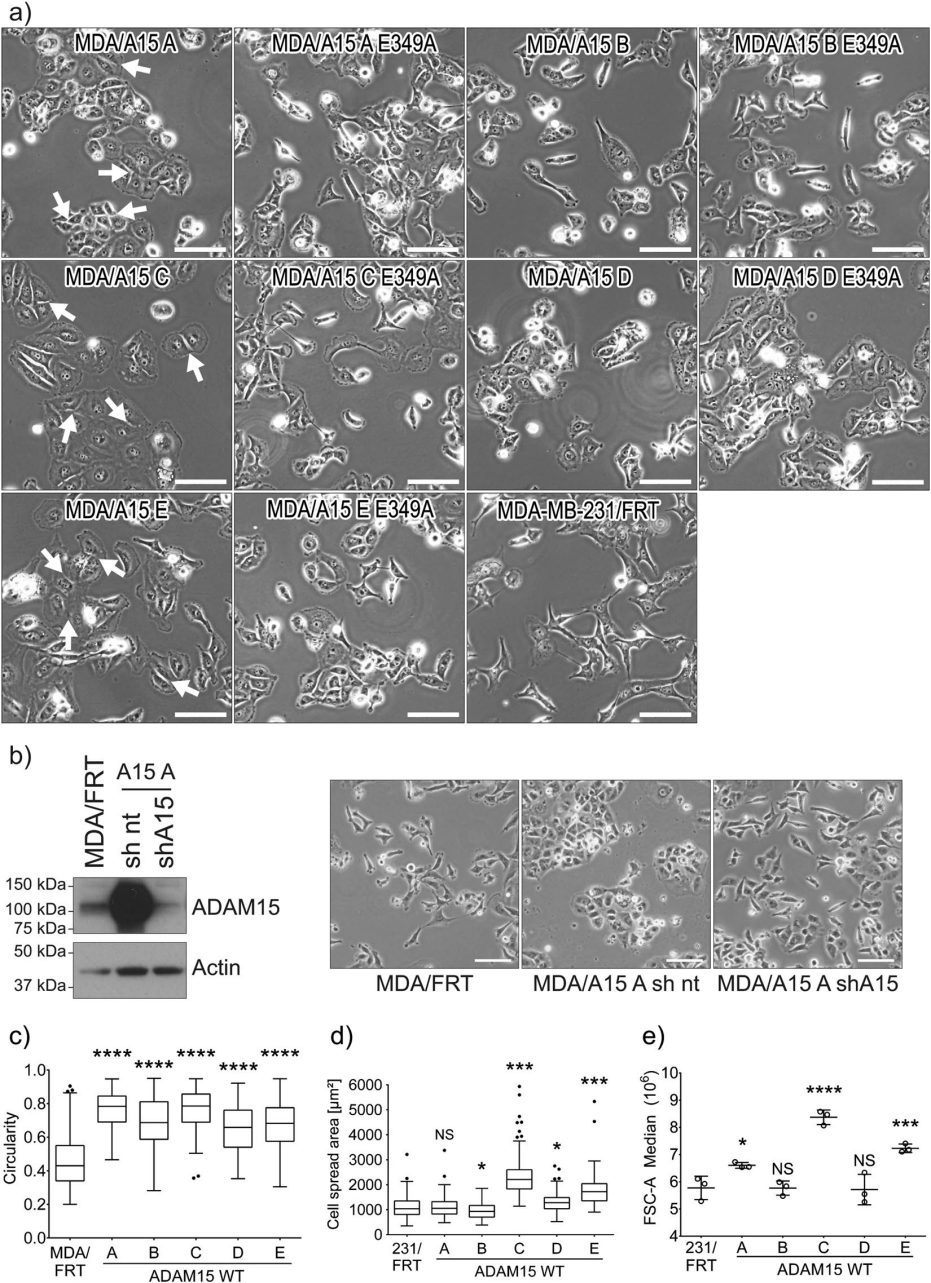
Expression of ADAM15 isoforms affects the morphology of breast cancer cells. (**a**) Phase-contrast images of ADAM15 isoform expressing MDA-MB-231 cells, Scale bar = 100 µm (**b**) downregulation of ADAM15 expression by shRNA to the coding region restores the morphology of MDA-MB-231 cells. Scale bar = 100 µm **c)** cell circularity (n = 150) and (**d**) cell spread area (n = 150) was quantified with ImageJ by analysing the outlines of cells in phase-contrast images. (**e**) Cell size measured by flow cytometry (n = 3). MDA/ADAM15 A-E WT expressing cells were compared to the MDA/FRT control cell line. Confidence intervals: ∗p ≤ 0.05, ∗∗p ≤ 0.01, ∗∗∗p ≤ 0.001, ∗∗∗∗p ≤ 0.0001.

The expression of ADAM15 isoforms led to morphological changes in MDA-MB-231 cells (Fig. 2). In general, we observed a tendency of cell clustering of varying degree with most wild type (WT) ADAM15 isoforms (Fig. 2a), with the exception of ADAM15B. This clustering effect was most prominent for ADAM15A expressing cells, which grew in epithelial-like islands. To further examine these observed morphological changes were due to ADAM15, we introduced shRNA targeting the coding region of ADAM15 into MDA-MB-231/ADAM15A cells, which resulted in substantial suppression of ADAM15 expression and reverted the epithelial-like phenotype attributable to ADAM15A-expression (Fig. 2b). Additionally, overexpression of all WT ADAM15 isoforms increased cell circularity (Fig. 2c), with the expression of ADAM15C and E isoforms resulting in an increase in cell spreading and size (Fig. 2d,e). In the MCF7/ADAM15 cell panel there were less dramatic morphological changes, as all of the lines grew in a similar fashion to parental MCF7 cells (Supplementary Fig. 2). However, there were subtle changes in that ADAM15D expressing MCF7 cells were smaller than all others, and ADAM15E MCF7 cells had altered cell-cell junctions with visible gapping between the cells, bridged by membrane projections, apparent in the higher magnification images in Supplementary Fig. 2.

**Figure 3:**
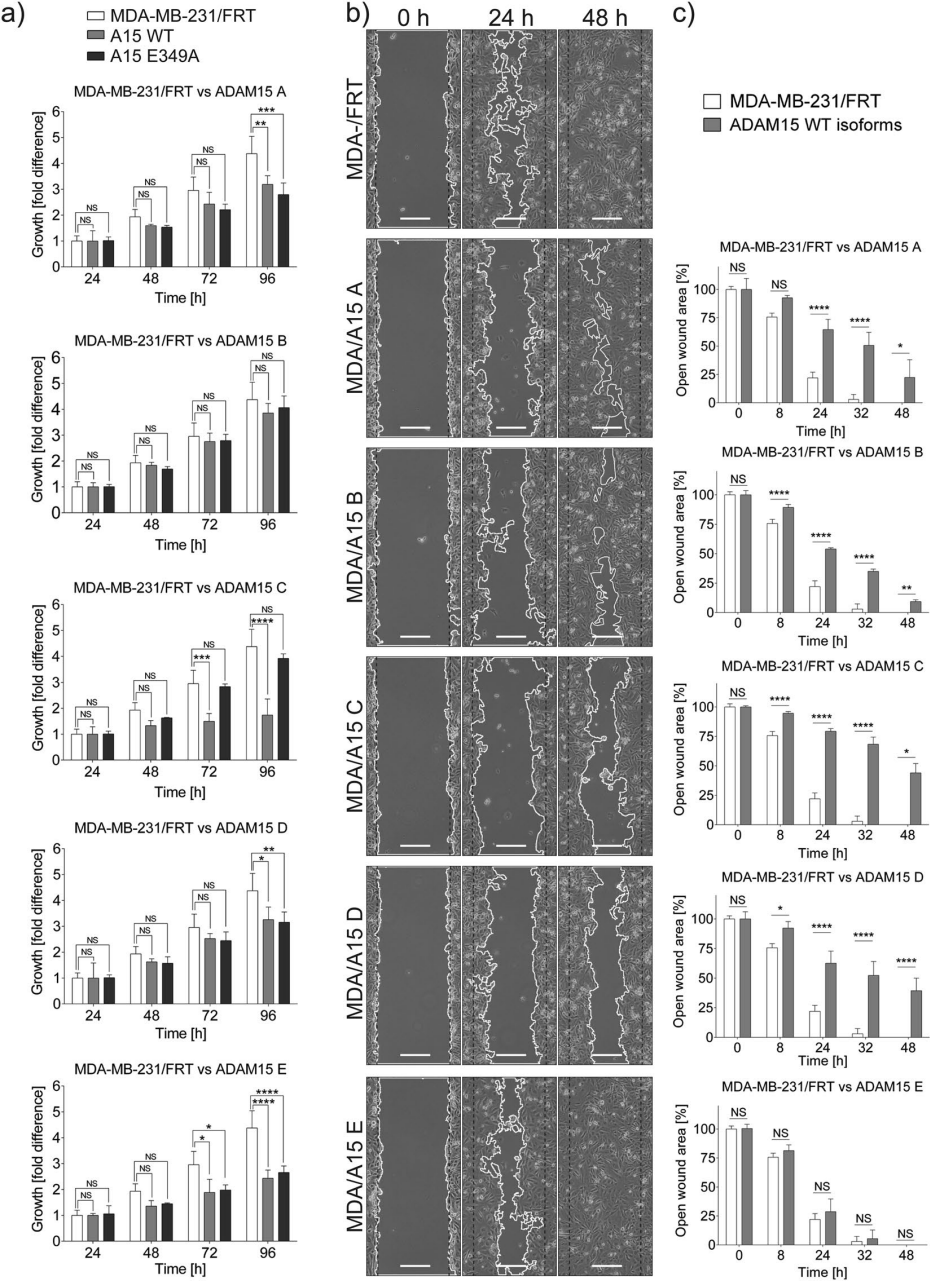
Expression of ADAM15 isoforms affects the proliferation rate and motility of MDA-MB-231 cells. (**a**) ADAM15 isoform expressing cells were seeded in 96 well plates at 3 × 10^3^ density in triplicates. CellTiter 96 Aqueous One solution (Promega) was added at 24, 48, 72, and 96 h, and absorbance measured at 450 nm. Values for 24 h time point were standardised to 1. Statistical significance was assessed with two-way ANOVA and Bonferroni post-test. Confidence intervals: *p < 0.05; **p < 0.01; ***p < 0.001; ****p < 0.0001; n = 3. (**b**) Representative images of scratch-wound assay at 0, 24 and 48 h. Scale bar = 200 µm. (**c**) Statistical analysis of the scratch wound assay comparing the motility of each ADAM15 isoform with parental MDA-MB-231 cells. Statistical significance was assessed with two-way ANOVA and Bonferroni post test. Confidence intervals: *p < 0.05; **p < 0.01; ***p < 0.001; ****p < 0.001; n = 3.

Focussing on the MDA-MB-231 cell panel, we tested whether ADAM15 isoforms may affect cell viability and the potential requirement for proteolytic activity by comparing the growth rates of the cells expressing WT and catalytically inactive ADAM15 isoforms using an MTS assay (Fig. 3a). The expression of ADAM15A, D and E WT as well as their catalytically inactive E349A counterparts reduced the growth rate of MDA-MB-231 cells at 96hrs, while the expression of ADAM15C reduced the growth rate of MDA-MB-231 cells in a catalytic function-dependent manner. The expression of ADAM15B (WT or inactive) had no effect on growth rate. Additional Western blot analysis confirmed that ADAM15 isoforms were processed to their respective active forms in these cells (Suppl. Fig. 1C). Comparison of the growth rate of MCF7 cells expressing ADAM15 isoforms did not reveal any significant differences, highlighting the relevance of cell context for the effects of ADAM15 (data not shown).

Previously it has been shown that ADAM15 either reduces or increases cell motility depending on the cell type^14–17^. To evaluate the influence of the individual ADAM15 isoforms on cell motility in the MDA-MB-231/FRT panel, we performed scratch wound assays (Fig. 3b,c). The ADAM15 isoforms, with the exception of ADAM15E, reduced healing of the cell monolayers significantly.

In summary, the morphological changes observed in MDA-MB-231 and MCF7 ADAM15 expressing cells were catalytic function-dependent, while the reduction in the growth rate of MDA-MB-231 cells depended on the catalytic activity of only ADAM15C isoform expressing cells.

### ADAM15 isoform-dependent up-regulation of Claudin-1 in breast cancer cells

To test whether ADAM15-dependent changes in cell morphology involved reversal of the mesenchymal phenotype of the MDA-MB-231 cells to a more epithelial phenotype, we analysed the expression of cell-cell junction molecules and Epithelial Mesenchymal Transition (EMT) markers. Expression of vimentin, slug, or E-cadherin (Supplementary Fig. 3) were unchanged in WT ADAM15A and ADAM15A E349 mutant expressing cells, demonstrating that ADAM15A does not cause mesenchymal-epithelial transition in MDA-MB-231 cells.

**Figure 4:**
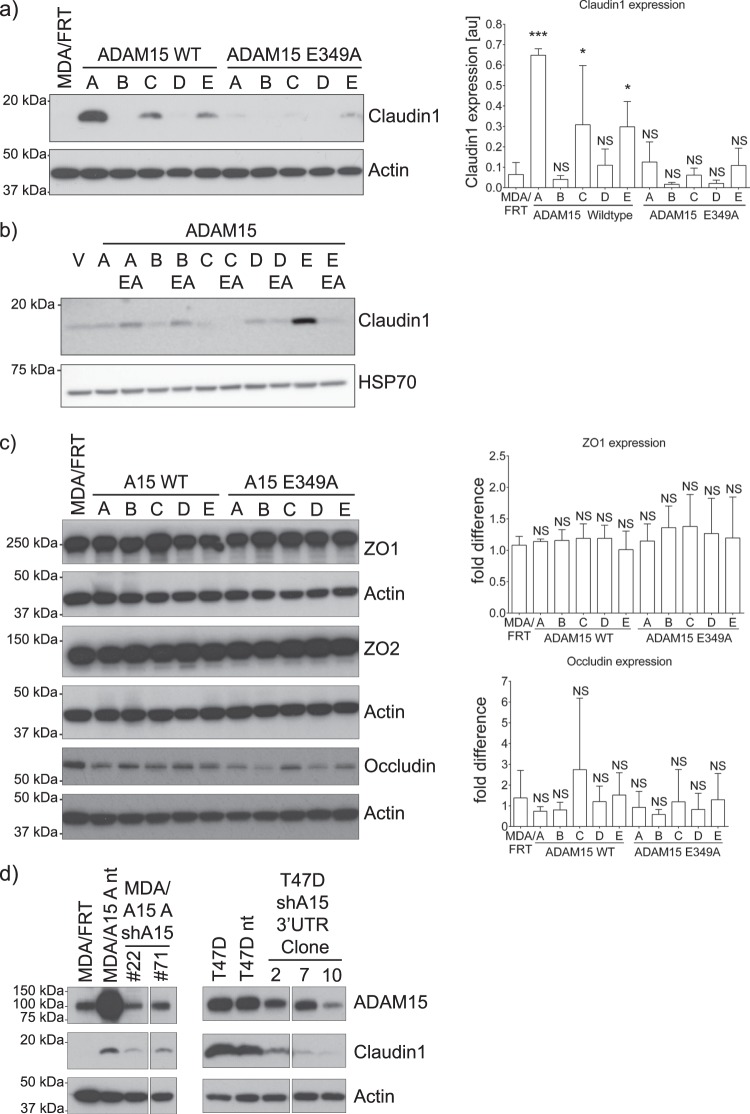
Expression of some ADAM15 isoforms leads to upregulation of claudin-1 in breast cancer cells. (**a**) Total protein extracts of MDA-MB-231 cells (n = 3) and (**b**) Total protein extracts of MCF-7 cells expressing ADAM15 isoforms grown to near-confluence were analysed for the expression of claudin-1. (**c**) Expression of the tight junction proteins ZO1 (n = 3), ZO2 (n = 1) and occludin (n = 5) in MDA/ADAM15 isoform expressing cells. (**d**) Stable downregulation of ADAM15 either by an shRNA to the coding region in MDA-MB-231 ADAM15A expressing cells or by an shRNA to the 3′UTR in T47D cells leads to a decrease in claudin1 expression. Dunnett’s multiple comparison test was carried out to assess significance. Confidence intervals: *p < 0.05; **p < 0.01; ***p < 0.001.

However, analysis of tight junction proteins revealed significant upregulation of Claudin-1 expression in WT ADAM15A and, to some degree, in WT ADAM15C and E expressing MDA-MB-231 cells (Fig. 4a) and predominantly in WT ADAM15E expressing MCF7 cells (Fig. 4b). These changes were not apparent in cells expressing the catalytically inactive ADAM15 isoforms, indicating that metalloproteinase function is necessary for ADAM15-mediated induction of Claudin-1. We did not observe significant changes in the expression of other tight junction molecules such as ZO1, ZO2 and occludin in either cell panel (Fig. 4c and not shown). Taqman qPCR analysis of mRNA confirmed that Claudin-1 up-regulation is at the transcriptional level (Supplementary Fig. 4).

Next we sought to investigate if claudin-1 expression depends on ADAM15 overexpression. In ADAM15A-expressing MDA-MB-231 cells, shRNA-mediated knockdown of ADAM15A led to reduced expression of Claudin-1 compared to levels seen in control MDA/ADAM15A/sh-NT cells (Fig. 4d, left panel), indicating that ADAM15 is necessary for Claudin-1 up-regulation in MDA-MB-231 cells. To confirm this further we made use of T47D breast cancer cells that have high expression levels of endogenous ADAM15 isoforms A, C and E (Supplementary Fig. 5), as well as of Claudin-1. Introduction of shRNA targeting the 3′UTR of ADAM15 into T47D cells down-regulated endogenous ADAM15 expression, leading to a concomitant decrease in Claudin-1 levels confirming that ADAM15 is responsible for driving Claudin-1 expression in these breast cancer cells.

To test the potential role of the elevated Claudin-1 expression in response to ADAM15A over-expression in regulating transepithelial electrical resistance (TEER), paracellular permeability, or cell migration, we transfected ADAM15A-overexpressing cells with Claudin-1 shRNA or non-targeting controls. Supplementary Fig. 6a shows that significant reduction of Claudin-1 expression was achieved. Subsequently cells were used to measure TEER, paracellular permeability and migration using the scratch wound assay. With respect to TEER and paracellular permeability, no significant differences were observed between MDA-MB-231/ADAM15A/sh-nontarget cells compared with MDA-MB-231/ADAM15A/sh-ADAM15 or MDA-MB-231/ADAM15A/sh-Claudin-1 cells, or between T47D/sh-nontarget or T47D/sh-ADAM15 (Supplementary Fig. 6b). Likewise, comparison of wound healing rates between MDA-MB-231/ADAM15A/sh-nontarget and MDA-MB-231/ADAM15A/sh-Claudin-1 cells (Supplementary Fig. 6c) showed no difference in wound closure, suggesting Claudin-1 has no role in regulating the speed of cell migration in these cells.

### The PI3K/mTOR pathway is involved in upregulating Claudin-1 expression downstream of ADAM15

**Figure 5:**
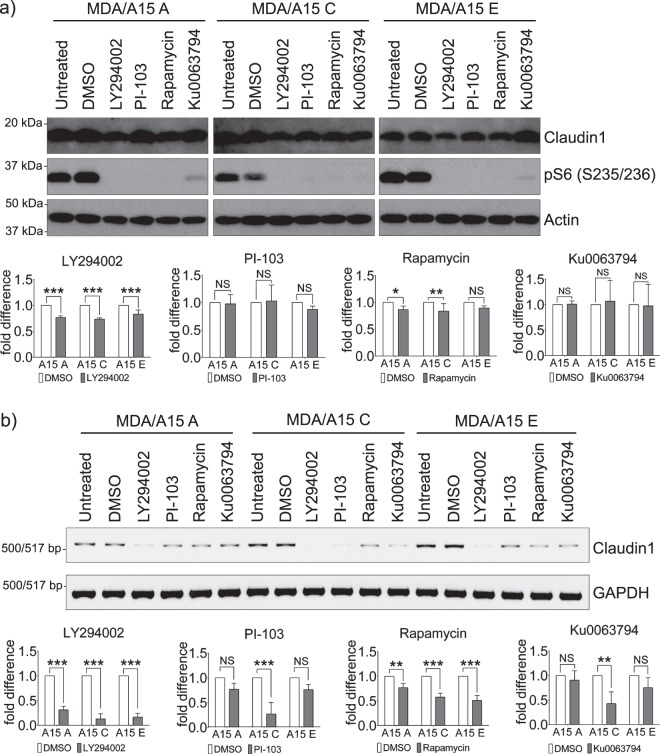
PI3K/mTOR signalling pathway is involved in upregulating claudin1 expression downstream of ADAM15 in MDA-MB-231 cells. Cells were treated with inhibitors of PI3K LY294002 (50 µM), PI-103 (1 µM), and of mTOR Rapamycin (100 nM) and Ku0063794 (1 µM) overnight. (**a**) Protein extracts were analysed for Claudin-1 expression. Statistical significance was assessed by two-way ANOVA. The inhibitor treatment was compared to the vehicle control for MDA/ADAM15 A, C and E expressing isoforms individually. Confidence intervals: ∗p < 0.05; ∗∗p < 0.01; ∗∗∗p < 0.001. n = 3 Phosphorylation of ribosomal protein S6 was used as a readout for PI3K/mTOR activity. (**b**) RT-PCR analysis of Claudin-1 expression. Quantification of Claudin-1 expression normalised with GAPDH. n = 3.

Several signalling pathways have been implicated in regulating Claudin-1 expression^18–22^. An initial screen of various pathway inhibitors allowed us to exclude PKC isoforms, Src family and MAPK involvement, while indicating a potential role for PI3K signalling pathway as a regulator of Claudin-1 expression downstream of ADAM15 (Supplementary Fig. 7). This prompted us to analyse the role of PI3K effector mTOR. Protein levels of Claudin-1 in MDA/ADAM15A, C and E expressing cells in response to two different specific pharmacological inhibitors of PI3K and mTOR showed moderate decreases (Fig. 5a), though cyclohexamide treatment indicates that Claudin-1 protein is stable for the duration of the assay in MDA/ADAM15A cells (Supplementary Fig. 8). The treatments did not affect ADAM15 expression (Supplementary Fig. 9). However, analysis of Claudin-1 mRNA levels following PI3K and mTOR inhibition confirmed their role in upregulating Claudin-1 expression downstream of ADAM15 (Fig. 5b). Additionally, PI3K inhibition by LY294002 for 24 hrs led to re-establishment of the spindle-like mesenchymal morphology in ADAM15A expressing cells as demonstrated in the phase-contrast images (Supplementary Fig. 10), while only moderated changes were observed in ADAM15C and E isoform expressing cells.

### ADAM15 co-localises with Claudin-1 and ZO1 at cell-cell junctions and is in a complex with ZO1

**Figure 6:**
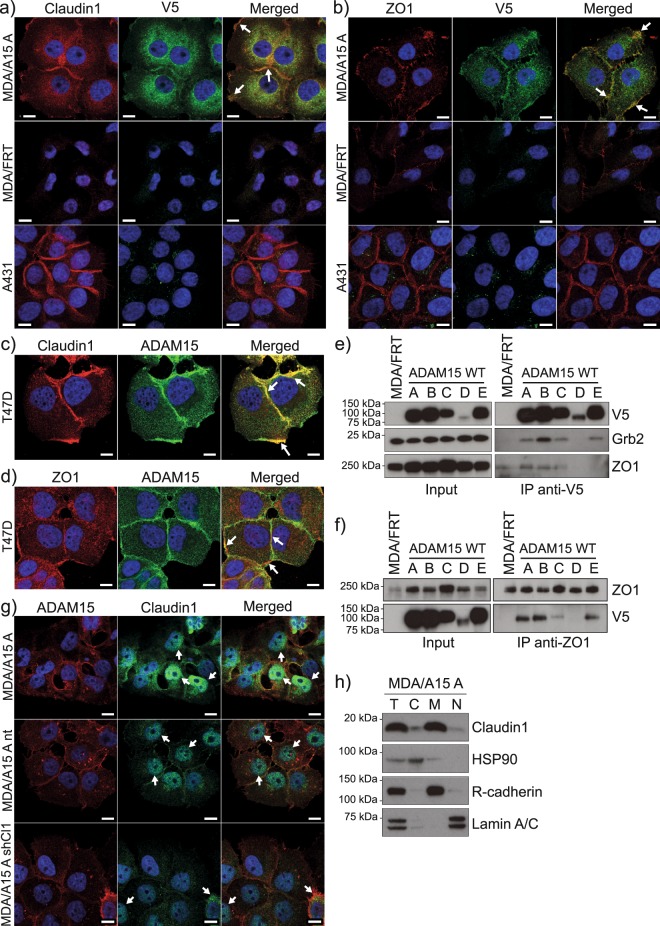
Complex formation between Claudin-1, ZO1 and ADAM15 in MDA-MB-231 and T47D cells. Co-localisation of overexpressed ADAM15 (V5, AlexaFluor-488, green) and (**a**) Claudin-1 (AlexaFluor-594, red) or (**c**) ZO1 (AlexaFluor-594, red) in MDA/ADAM15 A expressing cells. MDA/FRT and A431 cells do not contain V5 tagged ADAM15 and serve as negative control for V5. A431 were used as positive control for claudin-1cell-cell junction localisation. Co-localisation of ADAM15 (AlexaFluor-488, green) with (**c**) Claudin-1 (AlexaFluor-594, red) or (**d**) ZO1 (AlexaFluor-594, red) in T47D cells. Example areas of co-localisation are highlighted with arrows. Scale bar = 10 mm. Immunoprecipitation of (**e**) ADAM15 (V5) and (**f**) ZO1 from MDA/ADAM15 A-E expressing cells. V5 IP probed with V5, Grb2 (as a positive control for co-IP with ADAM15) and ZO1. ZO1 IP probed with ZO1 and V5. (**g**) Representative IF images of Claudin-1 localisation to nuclei in MDA/ADAM15A cells. ADAM15 (ADAM15-ECD, AlexaFluor-594, red) and claudin1 (AlexaFluor-488, green) localization in MDA/ADAM15A, MDA/ADAM15A/sh-non-target and MDA/ADAM15A/shClaudin1 expressing cells. Nuclei visualized by DAPI (blue). Scale bar = 10 µm. (**h**) Subcellular localization of claudin-1 by fractionation analysis of MDA/ADAM15A expressing cells. HSP90 serves as cytoplasmic, R-cadherin as membrane and Lamin A/C as nuclear control. T = Total cell lysate; C = cytoplasmic fraction; M = Membrane fraction; N = Nuclear fraction.

Aberrant expression and mis-localisation (cytoplasmic/nuclear instead of cell-cell junctions) of Claudin-1 is reported in several cancers, such as osteosarcomas and colorectal cancers^23,24^. Since ADAM15A isoform expressing cells grow in epithelial-like islands, we anticipated that Claudin-1 would be localised to cell-cell junctions. Immunofluorescence analysis (Fig. 6a) revealed that Claudin-1 is localised predominantly at cell-cell junctions, and at the cell periphery, and co-localises with ADAM15. Immunofluorescence analysis of endogenous Claudin-1 and ADAM15 in T47D breast cancer cells also demonstrated Claudin-1 and ADAM15 co-localisation at cell-cell junctions and the cell periphery (Fig. 6c). These data suggest that ADAM15 and Claudin-1 might be in a protein complex. ADAM15 ICD interacts with SH3 domain-containing cytoplasmic proteins via its proline-rich regions, and SH2 domain containing proteins via phosphorylated tyrosines^4,5,25^. Claudin-1 does not contain SH2 or SH3 interaction motifs, thus it is unlikely to interact directly with ADAM15. However, Claudin-1 contains a PDZ binding motif, with which it forms a complex with SH3 domain containing ZO1. Thus, ZO1 could potentially interact with ADAM15 and localise it close to Claudin-1. Indeed, immunofluorescence analysis of ADAM15A expressing MDA-MB-231 cells, as well as T47D cells with ZO1 and ADAM15 antibodies (Fig. 6b,d), demonstrated co-localisation of ADAM15 and ZO1 at cell-cell junctions, as well as the cell periphery in both cell lines. Claudin-1, as well as ZO1, demonstrated characteristic cell-cell junction localisation in A431 cells (Fig. 6a,b). Further immunoprecipitation analysis with either V5 (Fig. 6e) or ZO1 (Fig. 6f), or ZO2 (Supplementary Fig. 11), revealed that ZO1, ZO2 and all but one ADAM15 isoform (ADAM15D) co-immunoprecipitate, suggesting that ZO1 and ZO2 are likely interacting partners of ADAM15. The lack of co-IP between ADAM15D (that does not contain proline-rich motifs) and ZO1/2 suggests that ZO1/2-ADAM15 interaction is likely to be mediated via the SH3 domain of ZO1/2 and proline-rich regions in ADAM15 ICD.

In the course of Claudin-1 IF analysis small proportion of MDA-MB-231/ADAM15A cells showed positive staining for Claudin-1 in the nuclei as shown in Fig. 6g, while there was no appreciable Claudin-1 staining in MDA-MB-231/ADAM15A/shClaudin-1 cells. Subcellular fractionation of cells grown to confluency demonstrated that Claudin-1 is present not only in the membrane fraction, but low levels were detectable in the cytoplasmic as well as nuclear fractions (Fig. 6h).

## Discussion

Elevated expression of ADAM15 has been observed in numerous cancer types including breast^5,26,27^, prostate^27–29^, bladder^30^ and lung^31^. Indeed *ADAM15* has recently been identified in a large genome-wide association study of over 60,000 cancers in multiple sites as a locus with pleiotropic associations with breast and lung cancer^32^. In breast cancer, particular ADAM15 variants that result from alternative splicing of exons encoding the ICD have different functional consequences in relation to patient outcomes and shedding of FGFR2iiib^5,12^. How individual ADAM15 isoforms contribute to contrasting breast cancer prognosis has been unclear so far. To better understand the functional consequences of the overexpression of each isoform, we generated isogenic cell panels expressing individual ADAM15 isoforms in their wild type and catalytically inactive forms. The studies reported here reveal ADAM15 variant-specific effects on cell morphology, size, spread, as well as proliferation and migration. Moreover, the investigation also revealed ADAM15 variant-specific and catalytic activity-dependent upregulation of the expression of the tight junction protein Claudin-1, which may help to explain some aspects of the actions of ADAM15 in breast cancer.

ADAM15 isoform expression led to changes in the growth rate of MDA-MB-231 cells. The expression of all but ADAM15B isoform led to reduction in the growth rate of these cells. However, the catalytic activity of only ADAM15C isoform was necessary for the observed reduction. ADAM15 isoform expression led to changes in the motility of MDA-MB-231 cells. Overexpression of ADAM15 A-D isoforms reduced the motility of these cells over the analysed 48 hr period. This is unlikely to be the result of reduced proliferation, as the decrease in the migration rate was significantly apparent within 8hrs (ADAM15 B and C) and 24hrs (ADAM15 A and D) of monitoring, while there are no obvious changes in the proliferation rate in the first 48hrs of monitoring. ADAM15D overexpression had the most pronounced effect, with the wounds not closing up to 96hrs (data not shown). On the other hand, overexpression of ADAM15E had no effect on the motility. Detailed analysis of how ADAM15 isoforms effect cell motility are currently underway in our group.

Claudin-1 upregulation downstream of ADAM15 depends on the catalytic activity of ADAM15, as the expression of catalytically inactive E349A isoforms do not lead to significant Claudin-1 expression. Given that ADAM15 has been reported as an ectodomain sheddase for a variety of transmembrane proteins including EGFR ligands^8^, E-cadherin^9^, CD23^7^, MICB^10^, FGFR2iiib^12^, we hypothesise that ADAM15 isoforms shed distinct membrane tethered ligands that activate their cognate transmembrane receptors, activating intracellular signalling pathways affecting gene expression. We screened a panel of pharmacological inhibitors to a number of intracellular signalling pathways that have either been implicated in regulating Claudin-1 expression, or the components have been shown to interact with ADAM15 ICD. This established that the PI3K/Akt pathway is required downstream of ADAM15 isoform action to mediate Claudin-1 upregulation, while PKC, MAPK and Src family tyrosine kinase activities were not essential.

The PI3K/Akt/mTOR pathway regulates a variety of cellular functions in cancer, such as cell growth, survival, proliferation. PI3K, as well as mTOR, are activated downstream of a number of cell surface receptors. While there is evidence that ADAM15 associates with the p85 regulatory subunit of PI3K^25^ and thus could potentially affect PI3K activity, we believe this is not the case here. Our data demonstrate that ADAM15-mediated Claudin-1 upregulation via PI3K and mTOR depends on the metalloproteinase catalytic function of ADAM15, as there is no significant expression of Claudin-1 in cells expressing the catalytically inactive ADAM15. This strongly suggests that certain ADAM15 isoforms activate cell surface receptors in an autocrine manner by increasing the availability of their corresponding ligands through shedding, which then results in PI3K and mTOR activation. We observed that the expression of ADAM15 A, C and E isoforms led to significant increase in cell size, as measured by FACS analysis, further supporting elevated PI3K/mTOR activity.

This also indicates that at least one common ADAM15A, C and E ICD interacting protein might regulate the catalytic activity and/or substrate choice of these isoforms. This is in agreement with our previous data where ADAM15B had enhanced proteolytic activity towards FGFR2iiib when interacting with and being phosphorylated by Src^12^. Furthermore, we observed that ADAM15C expression reduces the growth rate of MDA-MB-231 cells in a catalytic function dependent manner. This points that ADAM15C isoform sheds at least one other substrate, which is necessary for the proliferation of MDA-MB-231 cells. This may in part explain why breast cancer patients with ADAM15C overexpression have better survival rates^5^. So far, the functions of ADAM15 have been elusive since it has been difficult to identify its substrates. Identification of ADAM15 isoform-dependent shed substrates using approaches such as TMT-MS-TAILS^33,34^, will have significant implications for cancer patients, as well as for advancing our understanding of the role of *ADAM15* splicing for cancer biology and progression.

Claudin-1 is a barrier forming tight junction protein, its expression generally ensuring decreased permeability. There is conflicting evidence of the role of ADAM15 in regulating permeability in a number of studies. Earlier work^35,36^ proposed that ADAM15 functions as a cell adhesion molecule, enhancing cell-cell interactions to decrease permeability in NIH3T3 cells, and that the surface expression of ADAM15 in HUVECs can be driven by VE-cadherin, leading to localisation of ADAM15 at adherens junctions. This concurs with our data demonstrating ADAM15 presence in cell-cell adhesions. However, in MDA-MB-231 cells, ADAM15 localisation to cell-cell junctions was isoform-dependent, with ADAM15A isoform showing the most prominent junctional localisation, and ADAM15B isoform not localising to cell-cell junctions even in over-confluent cultures.

Sun *et al*.^15^ demonstrated a requirement for ADAM15 for endothelial hyper-permeability and increased neutrophil transmigration in response to thrombin exposure. This was independent of ADAM15 protease activity, but required signalling via Src and Erk1/2. Additionally, these authors showed ADAM15 to be necessary for LPS-induced endothelial hyper-permeability and neutrophil transmigration in the lung^37^. On the other hand, expression of miR-147b improved human lung endothelial barrier function by down-regulating ADAM15^38^. These studies support the notion that while constitutive expression of ADAM15 might not be a determinant of basal endothelial barrier properties, it plays an active role in mediating endothelial hyper-permeability and neutrophil transmigration under inflammatory conditions. However, these effects of ADAM15 on endothelial barrier permeability have not been linked to altering the expression of tight junction related proteins such as Claudin-1. Moreover, our data show that the expression of ADAM15 in either MDA-MB-231 or MCF7 cells did not lead to changes in cell monolayer permeability, and the measurement of trans-epithelial resistance did not show significant differences in MDA-MB-231 cells with or without ADAM15 or Claudin-1 expression. Thus ADAM15 appears to have different effects on endothelial versus epithelial permeability, which is unsurprising, considering its pleiotropic functions in different cells and tissues.

There is growing evidence that certain tight junction proteins have more than junctional functions, as their overexpression or mislocalisation can contribute to cancer progression by enhancing cell survival, invasion and metastasis (reviewed in Leech 2015^39^). Specifically, the increased expression of Claudin-1 in colon cancer correlated with metastasis and poor prognosis^40–42^, and with poor outcome in high grade invasive ductal carcinoma of the breast where it correlated with the molecular subtype^43^. Tamoxifen treatment of MCF7 cells has been shown to lead to Claudin-1 overexpression that antagonises apoptosis and promotes drug resistance^44^. Additionally, expression of Claudin-1 provided resistance to anoikis in gastric and colon cancer cells^45,46^, as well as resistance to apoptosis in nasopharyngeal carcinoma cells^47^. Moreover, upregulation of Claudin-1 in ovarian carcinoma effusions has been associated with poor survival^48^.

As ADAM15A expressing MDA-MB-231 cells maintain strong cell-cell adhesions, it is possible to hypothesize that the expression of ADAM15A may lead to enhanced collective cell migration. Although aberrant expression of Claudin-1 in cancers has been implicated in promoting collective cell migration^49^, the role of ADAM15A-mediated Claudin-1 overexpression in MDA-MB-231 cells in collective cell migration remains to be elucidated. Nevertheless, the stable silencing of Claudin-1 in these cells did not lead to the disassembly of cell-cell junctions, nor did it alter the speed of cell migration.

Interestingly, we observed that in a small proportion of MDA-MB-231/ADAM15A cells, that have not formed the epithelial-like islands, Claudin-1 was localised to nuclei. This is very much in agreement with Fortier *et al*.^49^, where the cells at the leading edge of the migratory sheets have nuclear and cytoplasmic Claudin-1 localisation, while in the following cells it is localised in the cell-cell junctions, maintaining cohesion. Nuclear Claudin-1 expression has also been observed in metastatic lesions in colon cancer^24^, in nasopharyngeal cancer cells^47^, osteosarcoma cells^23^, in follicular thyroid carcinoma cells and FTC metastasis^50^.

Claudin-1 is overexpressed in hepatocytes, and is one of the major homing receptors for Hepatitis C virus^51^. It is tempting to speculate that ADAM15 mediated Claudin-1 upregulation might have a role in defining the metastatic niche, enhancing breast cancer cell attachment to hepatocytes and metastasis to the liver. Interestingly, SW480 colon cancer cells engineered to overexpress Claudin-1 formed tumors at a significantly higher rate and caused multiple liver metastases compared with the control cells^24^.

Amongst the ADAM family, ADAM15 isoforms have the widest range of ICD interaction partners identified so far. It will be important to identify which particular intracellular interactions lead to substrate selection for the isoform-specific catalytic function of ADAM15. Here we identified that the ZO family proteins also form complexes with ADAM15. This is in line with a recent study identifying ZO1 as an interaction partner of ADAM12^52^. ADAM15 isoforms A, C and E co-localised with ZO1 as judged by immunofluorescence analysis. Additionally, all ADAM15 isoforms, except ADAM15D, and ZO1 and ZO2 co-immunoprecipitated. Together, this suggests that the ADAM15 interaction with ZO1/2 is likely to be direct, involving the proline-rich regions in ADAM15 and the SH3 domains in ZO proteins.

In summary, our data show that ADAM15 expression in breast cancer cells leads to upregulated expression of the tight junction protein Claudin-1, which involves activation of the PI3K/mTOR pathway. The increased Claudin-1 expression is ADAM15 isoform-specific and catalytic activity-dependent, as well as influenced by the cell background based on the differences we observed between MDA-MB-231 and MCF-7 cell lines. We confirmed the dependence of Claudin-1 on ADAM15 in T47D cells, which express high endogenous levels of both proteins. ADAM15 also co-localizes with Claudin-1 at cell-cell junctions, potentially via the association of ZO1/2 SH3 domains with the ADAM15 ICD. These associations provide new insights into the mechanisms by which ADAM15 contributes to progression of breast and other cancers, highlighting in particular the significant differences between the ICD variant forms of ADAM15.

## Supplementary information

Supplementary information

## Data Availability

All data generated or analysed during this study are included in this published article [and its Supplementary Files].

## References

[1] Murphy Gillian (2008). The ADAMs: signalling scissors in the tumour microenvironment. Nature Reviews Cancer.

[2] Blobel CP (2005). ADAMs: key components in EGFR signalling and development. Nat Rev Mol Cell Biol..

[3] Edwards DR, Handsley MM, Pennington CJ (2009). The ADAM metalloproteinases. Mol Aspects Med..

[4] Poghosyan Z (2002). Phosphorylation-dependent interactions between ADAM15 cytoplasmic domain and Src family protein-tyrosine kinases. J Biol Chem..

[5] Zhong JL (2008). Distinct functions of natural ADAM-15 cytoplasmic domain variants in human mammary carcinoma. Mol Cancer Res..

[6] Kleino I, Ortiz RM, Huovila A-PJ (2007). ADAM15 gene structure and differential alternative exon use in human tissues. BMC Mol Biol..

[7] Fourie AM, Coles F, Moreno V, Karlsson L (2003). Catalytic activity of ADAM8, ADAM15, and MDC-L (ADAM28) on synthetic peptide substrates and in ectodomain cleavage of CD23. J Biol Chem..

[8] Schäfer B, Marg B, Gschwind A, Ullrich A (2004). Distinct ADAM metalloproteinases regulate G protein-coupled receptor-induced cell proliferation and survival. J Biol Chem..

[9] Najy AJ, Day KC, Day ML (2008). The ectodomain shedding of E-cadherin by ADAM15 supports ErbB receptor activation. J Biol Chem..

[10] Duan X, Mao X, Sun W (2013). ADAM15 is involved in MICB shedding and mediates the effects of gemcitabine on MICB shedding in PANC-1 pancreatic cancer cells. Mol Med Rep..

[11] Maretzky T (2009). Characterization of the catalytic activity of the membrane-anchored metalloproteinase ADAM15 in cell-based assays. Biochem J..

[12] Maretzky T (2009). Src stimulates fibroblast growth factor receptor-2 shedding by an ADAM15 splice variant linked to breast cancer. Cancer Res..

[13] Arakawa Y, Saito S, Yamada H, Aiba K (2009). Simultaneous treatment with camptothecin and valproic acid suppresses induction of Bcl-XL and promotes apoptosis of MCF-7 breast cancer cells. Apoptosis..

[14] Charrier L (2005). ADAM-15 inhibits wound healing in human intestinal epithelial cell monolayers. Am J Physiol - Gastrointest Liver Physiol..

[15] Sun C (2010). ADAM15 regulates endothelial permeability and neutrophil migration via Src/ERK1/2 signaling. Cardiovacular Res..

[16] Chen Q (2008). ADAM15 suppresses cell motility by driving integrin alpha5beta1 cell surface expression via Erk inactivation. Int J Biochem Cell Biol..

[17] Dong DD, Zhou H, Li G (2015). ADAM15 targets MMP9 activity to promote lung cancer cell invasion. Oncol Rep..

[18] Banan A (2005). Theta Isoform of protein kinase C alters barrier function in intestinal epithelium through modulation of distinct claudin isotypes: a novel mechanism for regulation of permeability. J Pharmacol Exp Ther..

[19] Iitaka D, Moodley S, Shimizu H, Bai X-H, Liu M (2015). PKCδ–iPLA2–PGE2–PPARγ signaling cascade mediates TNF-α induced Claudin 1 expression in human lung carcinoma cells. Cell Signal..

[20] Yoon CH (2010). Claudin-1 acts through c-Abl-protein kinase C delta (PKC delta) signaling and has a causal role in the acquisition of invasive capacity in human liver cells. J Biol Chem..

[21] Gan H, Wang G, Hao Q, Jane Wang Q, Tang H (2013). Protein kinase D promotes airway epithelial barrier dysfunction and permeability through down-regulation of claudin-1. J Biol Chem..

[22] Lee JY, Chiu Y-H, Asara J, Cantley LC (2011). Inhibition of PI3K binding to activators by serine phosphorylation of PI3K regulatory subunit p85 Src homology-2 domains. Proc Natl Acad Sci..

[23] Jian Y, Chen C, Li B, Tian X (2015). Delocalized Claudin-1 promotes metastasis of human osteosarcoma cells. Biochem Biophys Res Commun..

[24] Dhawan P (2005). Claudin-1 regulates cellular transformation and metastatic behavior in colon cancer. J Clin Invest..

[25] Kleino I, Ortiz RM, Yritys M, Huovila APJ, Saksela K (2009). Alternative splicing of ADAM15 regulates its interactions with cellular SH3 proteins. J Cell Biochem..

[26] Lendeckel U (2005). Increased expression of ADAM family members in human breast cancer and breast cancer cell lines. J Cancer Res Clin Oncol..

[27] Kuefer R (2006). ADAM15 disintegrin is associated with aggressive prostate and breast cancer disease. Neoplasia..

[28] Lucas N, Day ML (2009). The role of the disintegrin metalloproteinase ADAM15 in prostate cancer progression. J Cell Biochem..

[29] Burdelski C (2017). Overexpression of the A Disintegrin and Metalloproteinase ADAM15 is linked to a Small but Highly Aggressive Subset of Prostate Cancers. Neoplasia (United States)..

[30] Hiles GL (2016). ADAM15 is functionally associated with the metastatic progression of human bladder cancer. PLoS One..

[31] Schütz A (2005). Expression of ADAM15 in lung carcinomas. Virchows Arch..

[32] Fehringer G (2016). Cross-cancer genome-wide analysis of lung, ovary, breast, prostate, and colorectal cancer reveals novel pleiotropic associations. Cancer Res..

[33] auf dem Keller U, Prudova A, Eckhard U, Fingleton B, Overall CM (2013). Systems-Level Analysis of Proteolytic Events in Increased Vascular Permeability and Complement Activation in Skin Inflammation. Sci Signal..

[34] Schilling O, Barré O, Huesgen PF, Overall CM (2010). Proteome-wide analysis of protein carboxy termini: C terminomics. Nat Methods..

[35] Herren B (2001). ADAM15 Overexpression in NIH3T3 Cells Enhances Cell–Cell Interactions. Exp Cell Res..

[36] Ham C (2002). ADAM15 Is an Adherens Junction Molecule Whose Surface Expression Can Be Driven by VE-Cadherin. Exp Cell Res..

[37] Sun C (2013). ADAM15 deficiency attenuates pulmonary hyperpermeability and acute lung injury in lipopolysaccharide-treated mice. Am J Physiol Lung Cell Mol Physiol..

[38] Chatterjee V (2014). MicroRNA-147b regulates vascular endothelial barrier function by targeting ADAM15 expression. PLoS One..

[39] Leech AO, Cruz RGB, Hill ADK, Hopkins AM (2015). Paradigms lost — an emerging role for over-expression of tight junction adhesion proteins in cancer pathogenesis. Ann Transl Med..

[40] Kinugasa T (2010). Increased claudin-1 protein expression contributes to tumorigenesis in ulcerative colitis-associated colorectal cancer. Anticancer Res..

[41] Huo Q (2009). Claudin-1 protein is a major factor involved in the tumorigenesis of colorectal cancer. Anticancer Res..

[42] Resnick MB, Konkin T, Routhier J, Sabo E, Pricolo VE (2005). Claudin-1 is a strong prognostic indicator in stage II colonic cancer: a tissue microarray study. Mod Pathol..

[43] Lu S (2013). Claudin expression in high-grade invasive ductal carcinoma of the breast: correlation with the molecular subtype. Mod Pathol..

[44] Akasaka H (2010). Anti-apoptotic effect of claudin-1 in tamoxifen-treated human breast cancer MCF-7 cells. BMC Cancer..

[45] Huang J (2014). Claudin-1 enhances tumor proliferation and metastasis by regulating cell anoikis in gastric cancer. Oncotarget..

[46] Singh AB, Sharma A, Dhawan P (2012). Claudin-1 expression confers resistance to anoikis in colon cancer cells in a Src-dependent manner. Carcinogenesis..

[47] Lee JW (2010). Upregulated claudin-1 expression confers resistance to cell death of nasopharyngeal carcinoma cells. Int J Cancer..

[48] Kleinberg L, Holth A, Trope CG, Reich R, Davidson B (2008). Claudin upregulation in ovarian carcinoma effusions is associated with poor survival. Hum Pathol..

[49] Fortier AM, Asselin E, Cadrin M (2013). Keratin 8 and 18 loss in epithelial cancer cells increases collective cell migration and cisplatin sensitivity through claudin1 up-regulation. J Biol Chem..

[50] Zwanziger D (2015). The impact of CLAUDIN-1 on follicular thyroid carcinoma aggressiveness. Endocr Relat Cancer..

[51] Zhu YZ, Qian XJ, Zhao P, Qi ZT (2014). How hepatitis C virus invades hepatocytes: The mystery of viral entry. World J Gastroenterol..

[52] Dekky B, Ruff M, Bonnier D, Legagneux V, Théret N (2018). Proteomic screening identifies the zonula occludens protein ZO-1 as a new partner for ADAM12 in invadopodia-like structures. Oncotarget..

