# Supplementary material for: ADAM15 mediates upregulation of Claudin-1 expression in breast cancer cells
Source: Sci Rep. 2019 Aug 29;9:12540. doi: 10.1038/s41598-019-49021-3 (PMC6715704; doi:10.1038/s41598-019-49021-3)

Supplementary information

ADAM15 mediates upregulation of Claudin-1 expression in breast cancer cells

Jens Mattern[1], Christian S Roghi[3*], Melanie Hurtz[1#], Vera Knäuper [2], Dylan R Edwards[3], Zaruhi Poghosyan[1]

[1] Division of Cancer and Genetics, School of Medicine, College of Biomedical and Life Sciences, Cardiff University Heath Park, Cardiff, CF14 4XN
[2] Oral and Biomedical Sciences, School of Dentistry, College of Biomedical and Life Sciences, Cardiff University, Heath Park, Cardiff, CF14 4XY
[3] School of Biological Sciences and Norwich Medical School, University of East Anglia, Norwich Research Park, Norwich, NR4 7TJ
* Current address: Quadram Institute Bioscience, Norwich Research Park, Norwich, Norfolk, NR4 7UA, UK
[#]Current address: MLM Medical Labs GmbH, Dohrweg 63, 41066 Mönchengladbach , Germany

Corresponding authors: **Zaruhi Poghosyan** (z.poghosyan@cardiff.ac.uk).
Division of Cancer and Genetics, School of Medicine, College of Biomedical and Life Sciences, Cardiff University Heath Park, Cardiff, CF14 4XN
Dylan R. Edwards (Dylan.edwards@uea.ac.uk)
School of Biological Sciences and Norwich Medical School, University of East Anglia, Norwich Research Park, Norwich, NR4 7TJ

Supplementary tables

Table 1: Antibodies used for western blot.

Antibody	Supplier	Catalogue number	Dilution
**Actin**	Sigma	A2066	1:1,000
**ADAM15 (ICD)**	Abcam	ab39159	1:2,000
**Claudin1**	Invitrogen	37-4900	1:1,000
**E-cadherin**	CST	#3195	1:1,000
**FAM190A**	Santa Cruz	sc-246676	1:500
**Grb2**	CST	#3972	1:1,000
**HSP70**	CST	#4872	1:1,000
**HSP90**	CST	#4874	1:1,000
**Lamin A/C**	CST	#4777	1:10,000
**Occludin**	Invitrogen	71-1500	1:3,000
**pS6 (S235/236)**	CST	#2211	1:1,000
**R-cadherin**	GeneTex	GTX62825	1:50,000
**Slug**	CST	#9585	1:1,000
**V5**	Invitrogen	46-0705	1:10,000
**Vimentin**	CST	#5741	1:5,000
**ZO1**	CST	#5741	1:1,000
**ZO2**	CST	#8193	1:1,000
**Mouse-HRP**	GE Healthcare	NA-931	-
**Rabbit-HRP**	GE Healthcare	NA-934	-

Table 2: Antibodies used for Immunofluorescence analysis.

Antibody	Supplier	Catalogue number	Dilution
**ADAM15 (ECD)**	R&D Systems	MAB935	1:100
**Claudin1**	Invitrogen	37-4900	1:100
**Claudin1**	Invitrogen	51-9000	1:100
**V5**	Invitrogen	46-0705	1:500
**ZO1**	CST	#8193	1:200
**Mouse-AF488**	Invitrogen	A11001	1:1,000
**Rabbit-AF488**	Invitrogen	A11008	1:1,000
**Mouse-AF568**	Invitrogen	A11031	1:1,000
**Rabbit-AF568**	Invitrogen	A11011	1:1,000

Table 3: Used inhibitors

Inhibitor	Supplier	Catalogue number	Concentration
Bim1	Merck/Millipore	203290	1 µM
FLLL31	Sigma	F9057	5 µM
Gö6976	Merck/Millipore	365250	1 µM
Ku0063794	Sigma	SML0382	1 µM
LY294002	CST	#9901	50 µM
PD98059	Sigma	P215	10 µM
PI-103	Selleckchem	S1038	1 µM
PP2	Sigma	P0042	100 nM
Rapamycin	Merck/Millipore	553210	100 nM
Rottlerin	Merck/Millipore	557370	5 µM
SB203580	Sigma	S8307	10 µM

Table 4: Mission shRNAs (Sigma) used for downregulation of gene expression.

Target	Catalogue number
ADAM15	NM 003815.3-1890s21c15
ADAM15	NM 003815.3-1361s21c1
ADAM15	NM 003815.3-1736s21c1
ADAM15	NM 003815.2-497s1c1
ADAM15	NM 003815.2-1076s1c1
CLDN1	NM 021101.3-902
CLDN1	NM 021101.3-402
CLDN1	NM 021101.3-305
CLDN1	NM 021101.3-626
CLDN1	NM 021101.3-627

Table 5: PCR Primer sequences.

Gene	Direction	Sequence
CLDN1	Forward	5'-ccaacgcggggctgcagctgttg-3'
CLDN1	Reverse	5'-ggatagggccttggtgttgggtaag-3'
GAPDH	Forward	5'-cgtcaaggctgagaacgggaagcttgtcatcaatgg-3'
GAPDH	Reverse	5'-catgccagtgagcttcccgttcagctcagggatg-3'

Supplementary Data

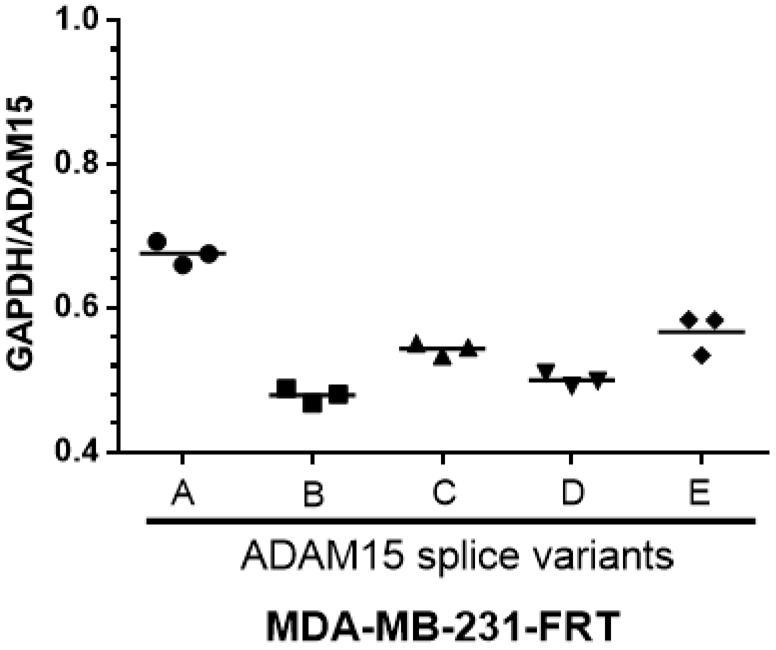

S. Figure 1a: MDA-MB-231-FRT cells express predominantly ADAM15 A isoform. ADAM15 splice profile was analysed by qPCR. GAPDH was used as endogenous control. GAPDH-Ct values were devided by Ct values for the targets and are expressed as GAPDH ratio

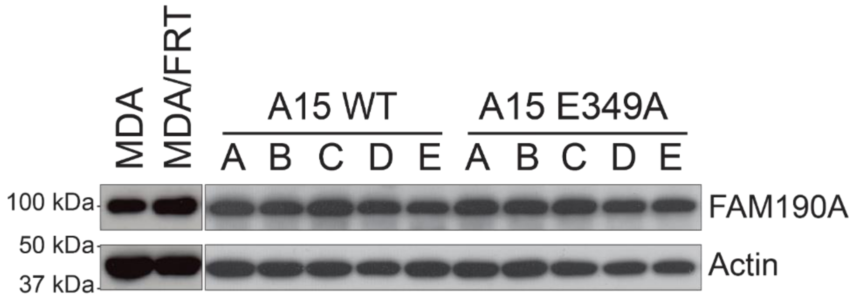

S. Figure 1b: Expression of FAM190A in ADAM15 isoform expressing MDA-MB-231 cells is not affected.

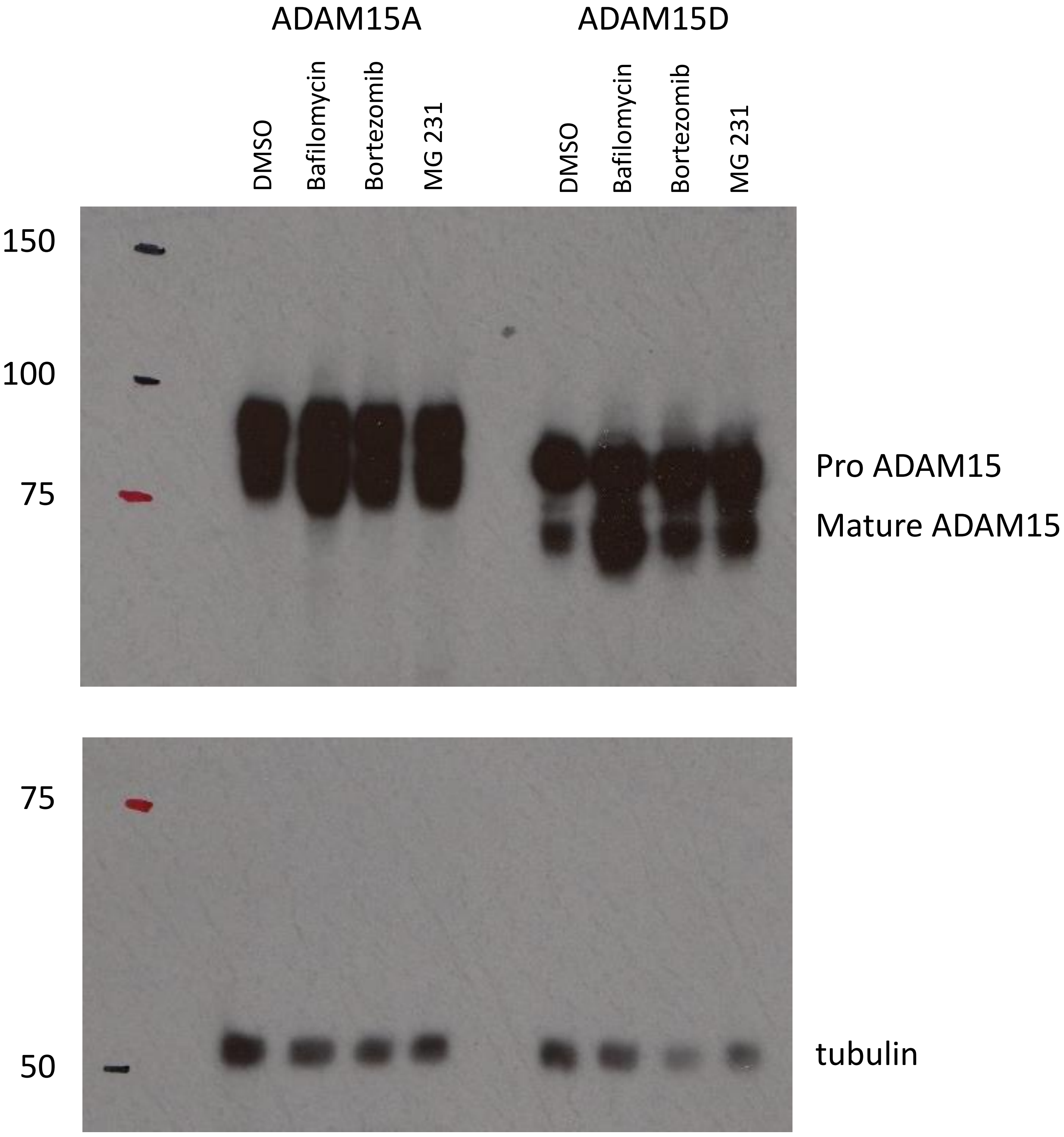

S. Figure 1c: ADAM15A and D expressing cells were treated with the indicated inhibitors for 24 hrs. Total lysates were separated on 7% SDS-PAGE, followed by immunoprobing with anti V5 (top panel) or anti-tubilin antibodies. The mature form of the protein accumulates when autophagy and proteasomal degradation are inhibited with bafilomycin

Supplementary Data

a) MCF-7/Vector MCF-7/ADAM15 A MCF-7/ADAM15 B

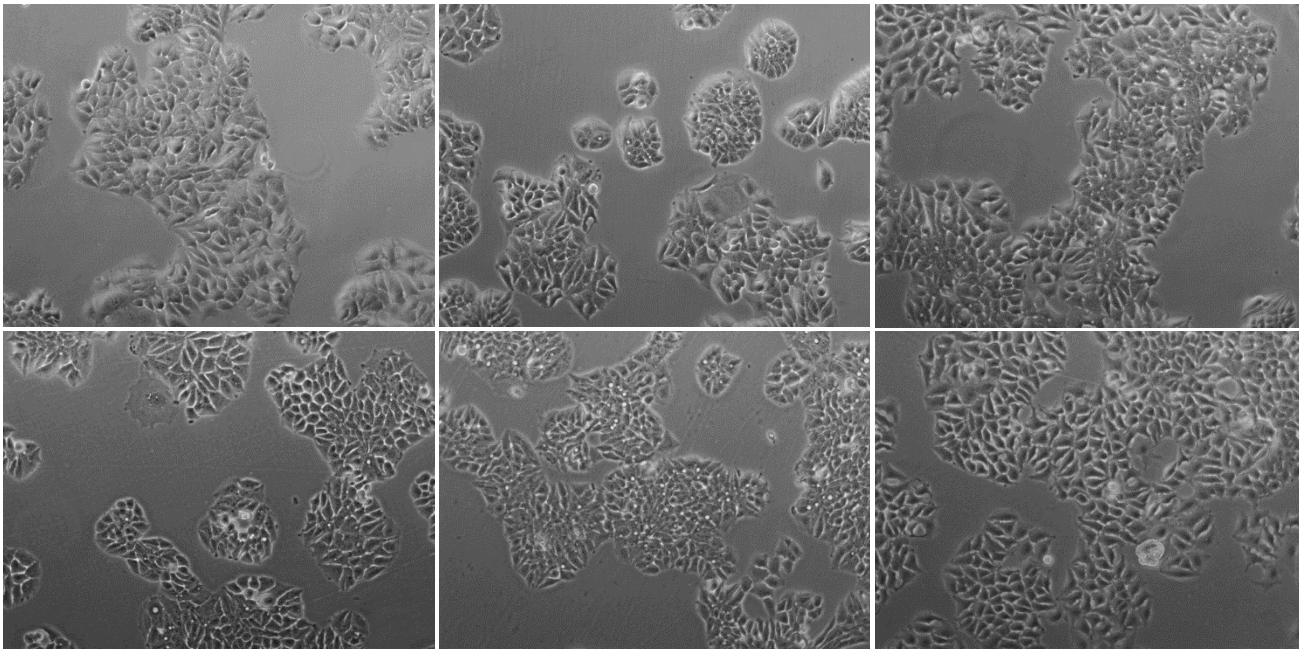

MCF-7/ADAM15 C MCF-7/ADAM15 D MCF-7/ADAM15 E

b) MCF-7/Vector MCF-7/ADAM15 E

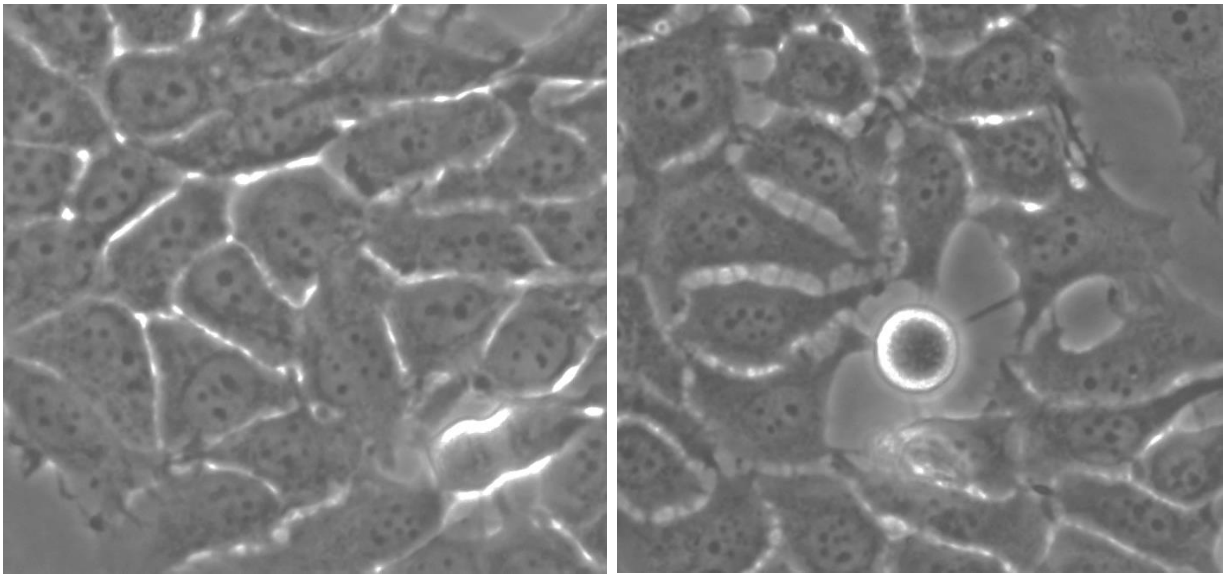

S. Figure 2: Expression of ADAM15 E isoform affects the morphology of MCF-7 cells . **a)** Phase-contrast images of ADAM15 isoform expressing MCF-7 cells. **b)** Magnified phase-contrast images of MCF-7/ADAM15 E expressing cells.

Supplementary Data

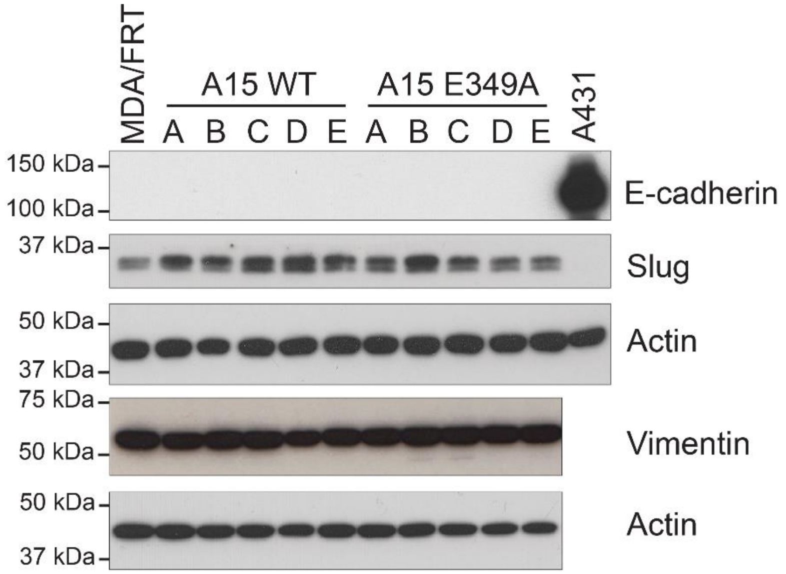

S. Figure 3: Total protein extracts from ADAM15 isoform expressing cells were analysed for the expression of the indicated EMT markers.

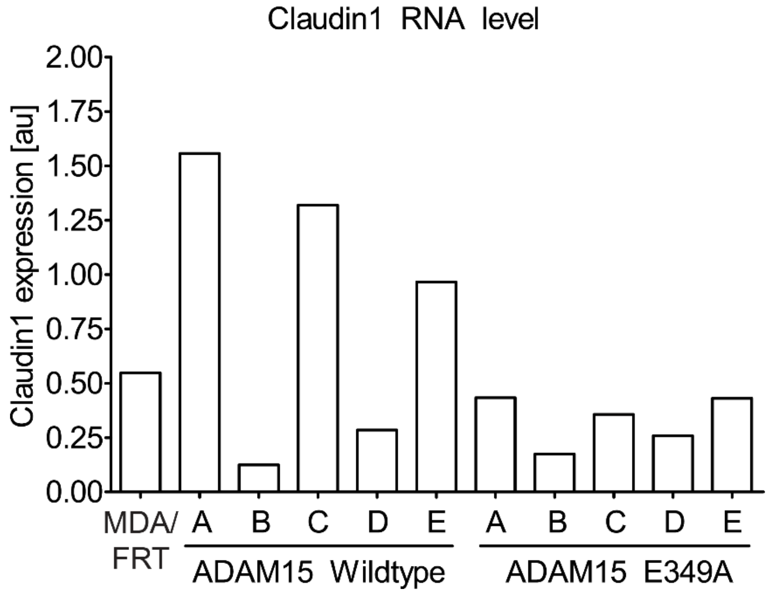

S. Figure 4: Taqman analysis of the RNA from ADAM15 isoform expressing MDA-MB-231 cells. n=1

Supplementary Data

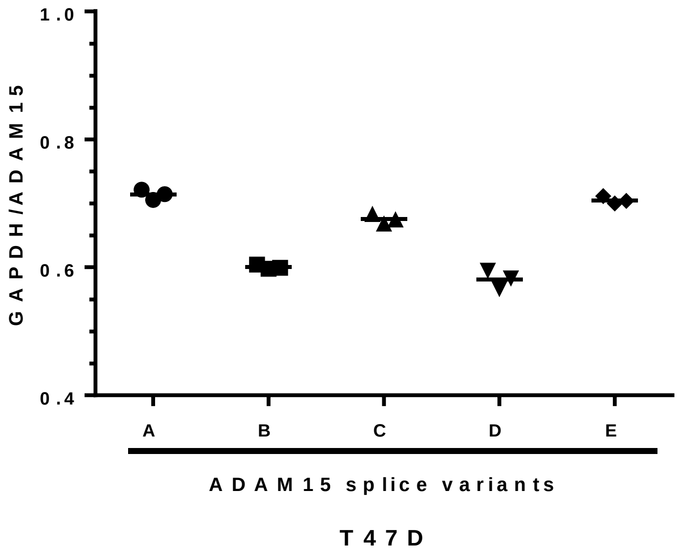

S. Figure 5. T47D cells express ADAM15 A, C, and E isoforms. ADAM15 splice profile was analysed by qPCR. GAPDH was used as endogenous control. GAPDH-Ct values were devided by Ct values for the targets and are expressed as GAPDH ratio.

Supplementary Data

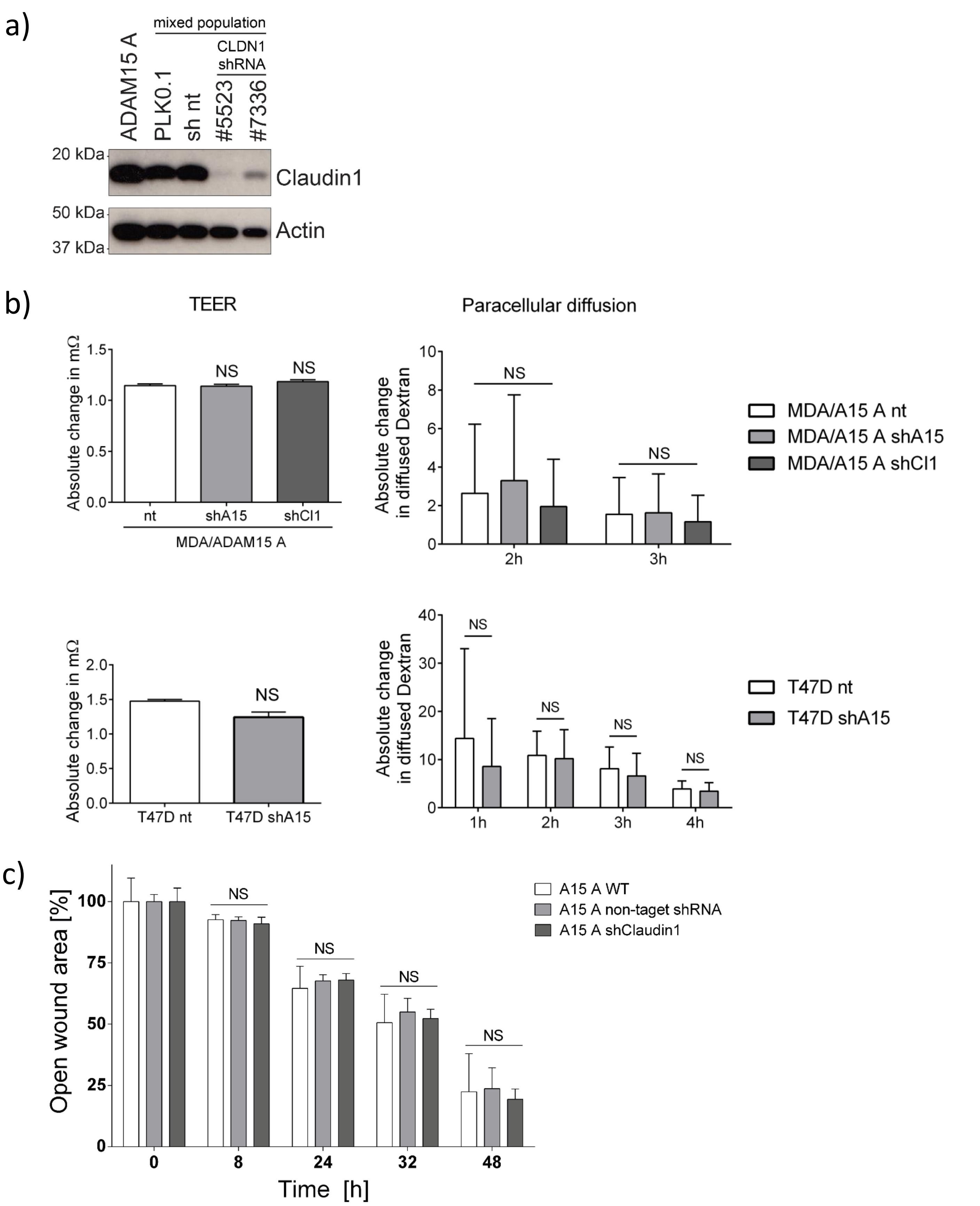

S. Figure 6: a) Stable downregulation of claudin1 by shRNA in MDA-MB-231 ADAM15 A expressing cells**. b)** Transepithelial electrical resistance (TEER) and paracellular diffusion (Para) measurement. TEER samples were compared to the nt control whearas the cell lines for para were compard to each other at each time point. **c)** Wound healing assay of MDA/ADAM15 A, MDA/ADAM15 A non-target shRNA and MDA/ADAM15 A shClaudin1 expressing cells. Bar chart comparing the wound closure of the cell lines.

Supplementary Data

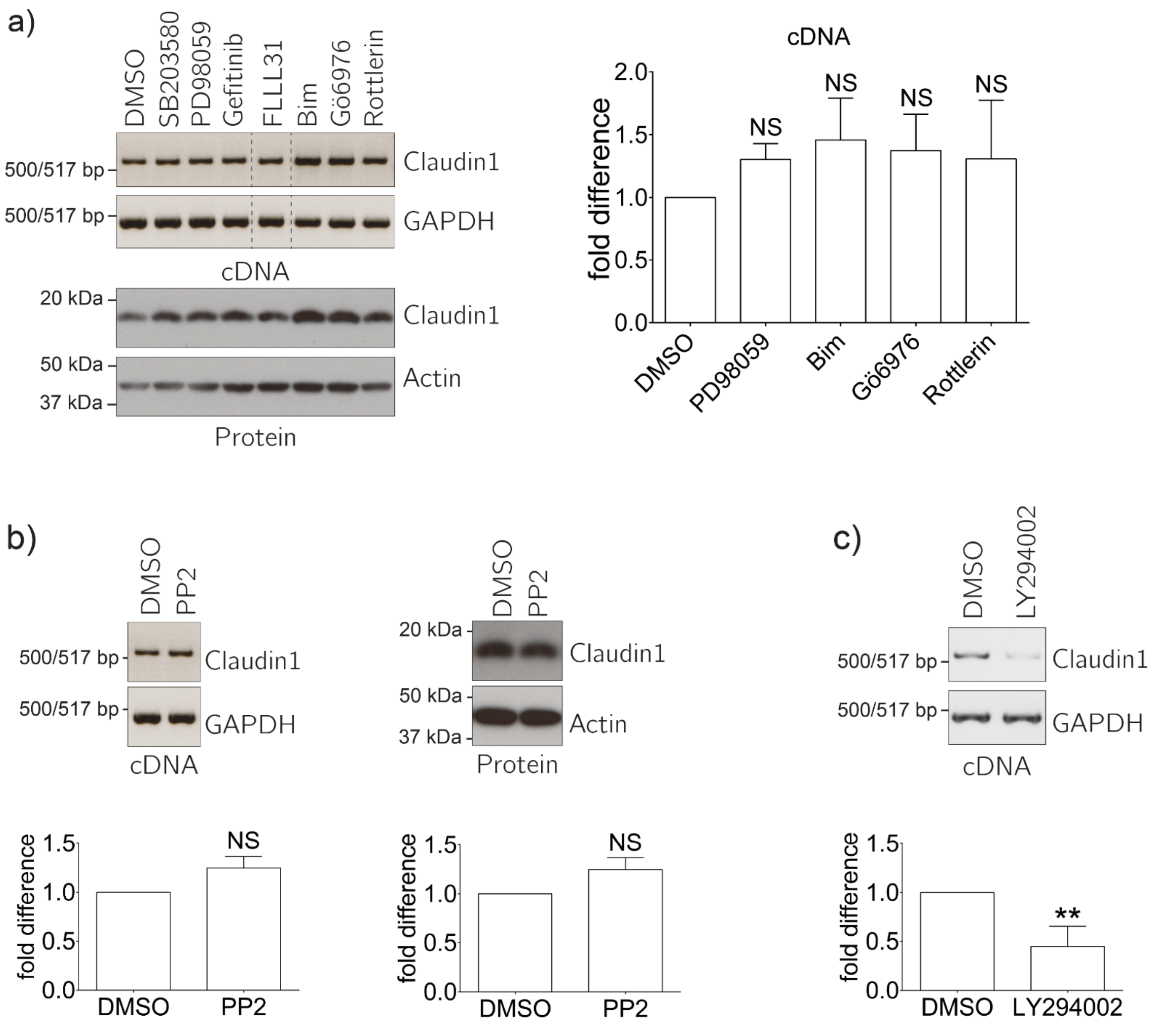

S Figure 7: Screening for signalling pathways potentially involved in claudin1 upregulation in MDA/ADAM15 A expressing cells. Duration of treatment was 24h with following inhibitors: SB203580 (10 mM; n=1), PD98059 (10 mM; n=4), Gefitinib (0.5 mM; n=2), FLLL31 (5 mM; n=1), Bim (1 mM; n=3), G¨o6976 (1 mM; n=3), Rottlerin (5 mM; n=3), PP2 (100 nM; n=3) and LY294002 (50 mM; n=5). Densitometry and analysis of the mRNA was performed using ImageJ. Claudin1/GAPDH ratio was normalised to the DMSO control. The result is shown as fold difference in bar graphs. To address statistical significance one-way ANOVA was performed. The inhibitor treated samples were compared to the vehicle control. Confidence intervals are as follows: ∗ = < 5 0.05; ∗∗ = p < 0.01; ∗∗∗ = p < 0.001. **a)** Representative images and quantification of analysed mRNA and protein. GAPDH was used as control for mRNA analysis whereas actin was used as control for protein. The RNA was reverse transcribed to cDNA and amplified with claudin1 and GAPDH specific primers followed by separation on a agarose gel. **b)** mRNA and protein analysis of Src family kinase inhibitor PP2 (100 nM). Claudin1/Actin ratio was normalized to the DMSO control. **c)** mRNA analysis of the PI3K inhibitor LY294002 (50 mM).

Supplementary Data

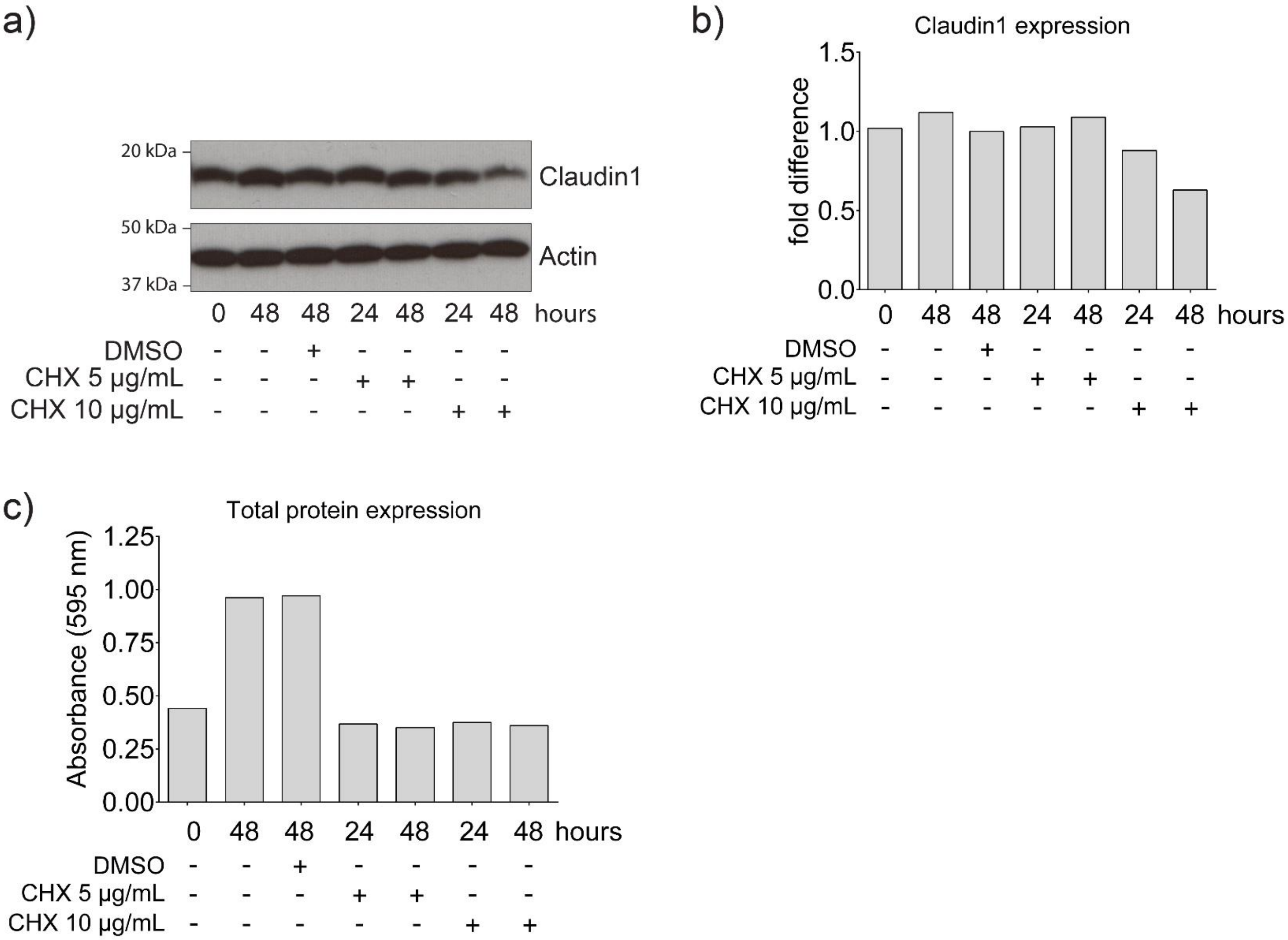

S Figure 8: Cycloheximide (CHX) treatment of MDA/ADAM15 A expressing cells after 0, 24 and 48h with either 5 µg/mL or 10 µg/mL. **a)** Claudin1 expression with and without CHX treatment. **b)** Densitometry of the a) normalised to actin. **c)** Total protein expression of the used samples determined by BCA protein quantification.

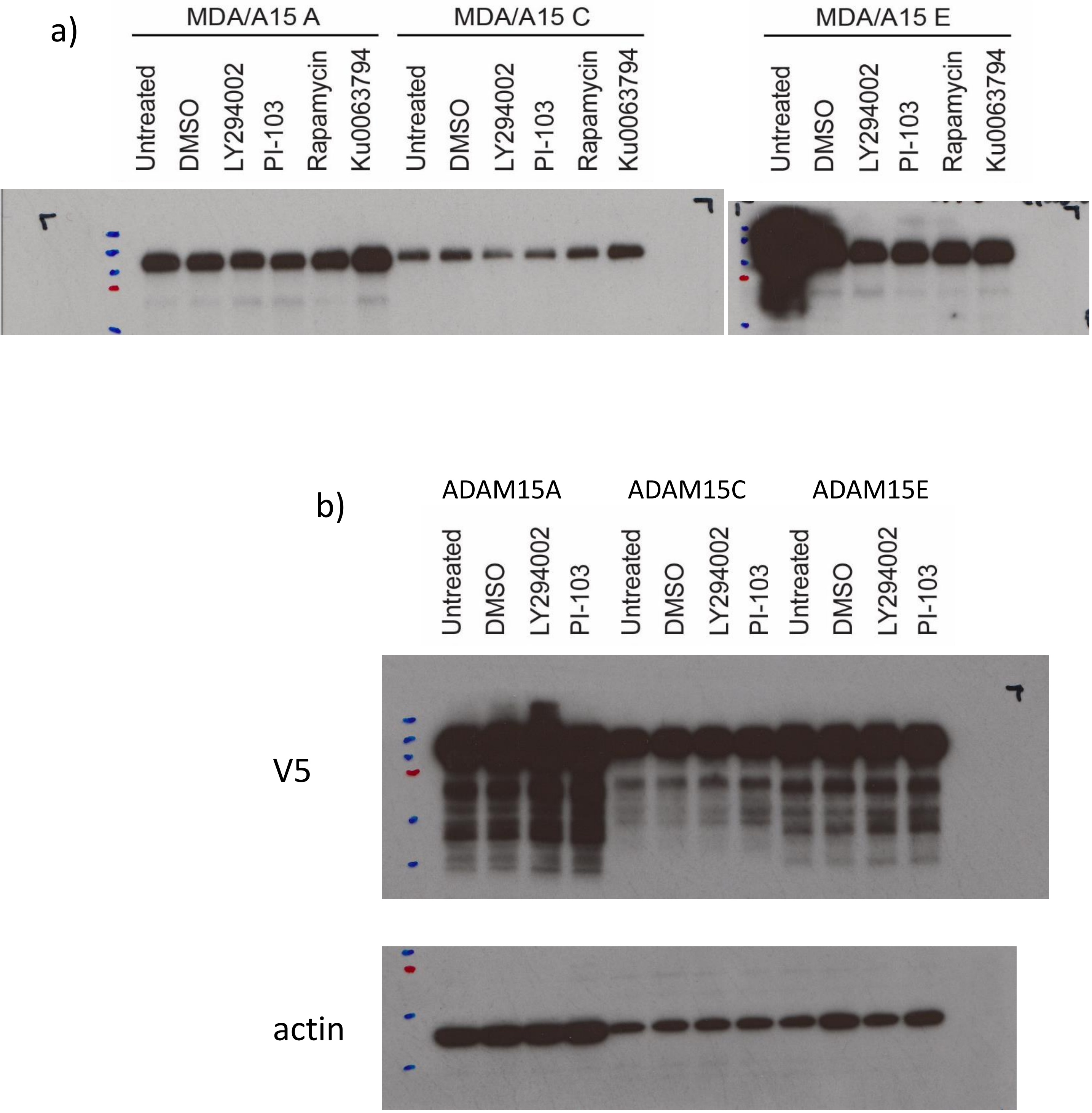

S Figure 9: a) WB analysis of V5-tagged ADAM15 expression in MDA-MB-231 cells in response to the indicated treatments to match the set presented in main Figure 5. Because of the artefact on the WB in the ADAM15E set, we cannot include this in the main Figure 5. **b)** WB analysis of V5-tagged ADAM15 expression in MDA-MB-231 cells in response to the indicated treatments in a smaller experiment.

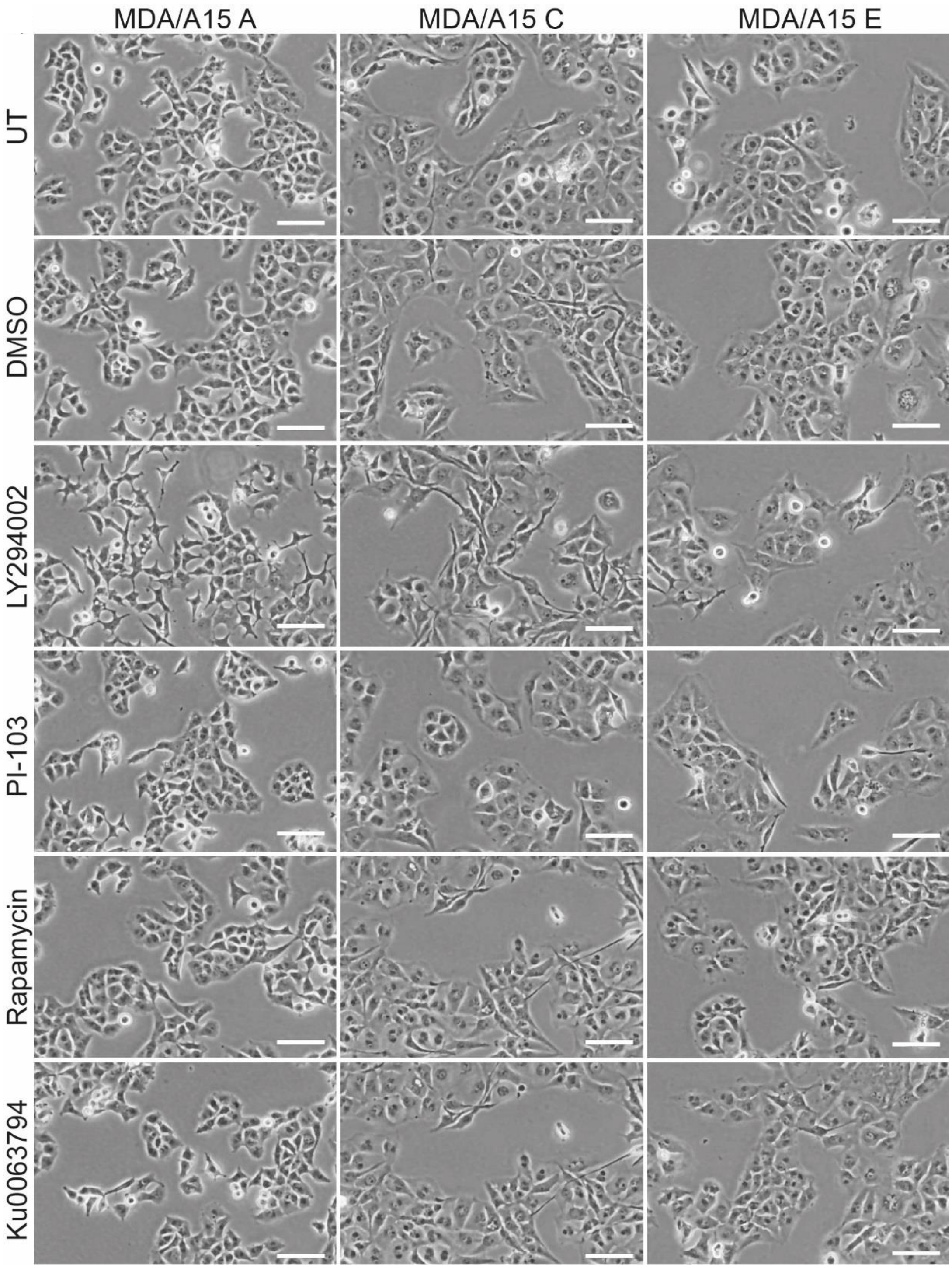

S Figure 10 Cells were treated with inhibitors of PI3K LY294002 (50 µM), PI-103 (1 µM), and of mTOR Rapamycin (100 nM) and Ku0063794 (1 µM) overnight. Representative phase-contrast images of MDA/ADAM15 A, C and E expressing cells after 24h of inhibitor treatment.

Supplementary Data

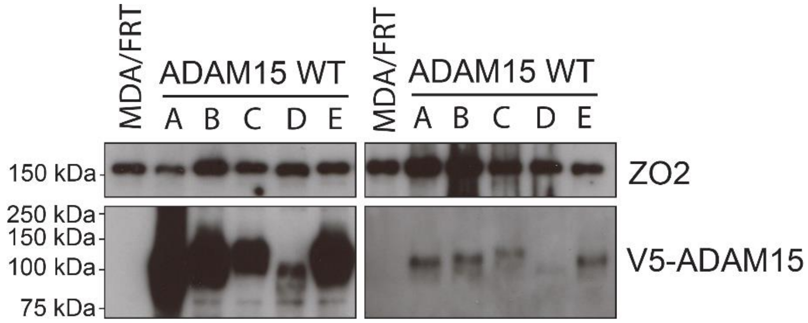

S. Figure 11 : Co-IP of ADAM15 and ZO2 in MDA/FRT and MDA/ADAM15 A-E expressing cells. n=2

Larger areas of the western blots used in the paper

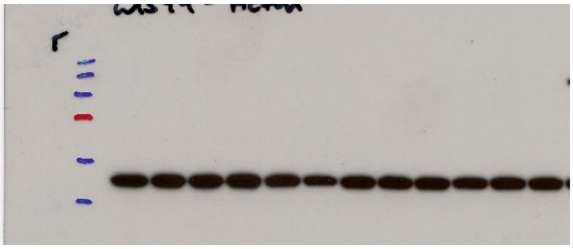

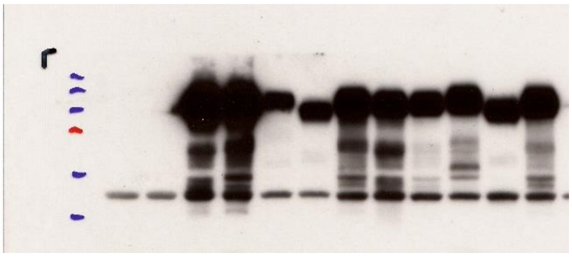

larger areas of the blots used in Fig 1b.
The top blot detecting actin was subjected to mild stripping and reprobed with V5 in the bottom.

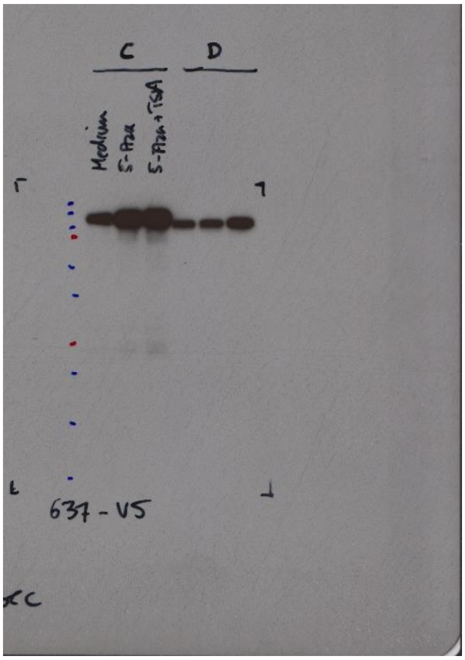

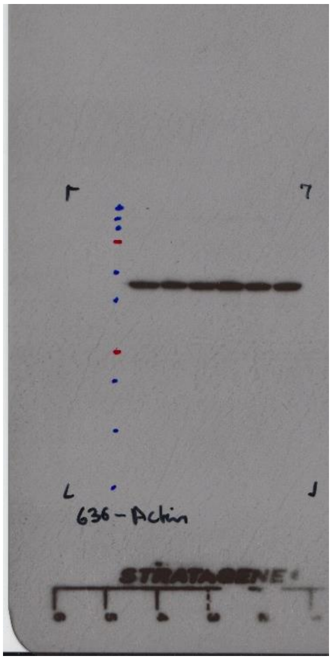

larger areas of the blots used in Fig 1c

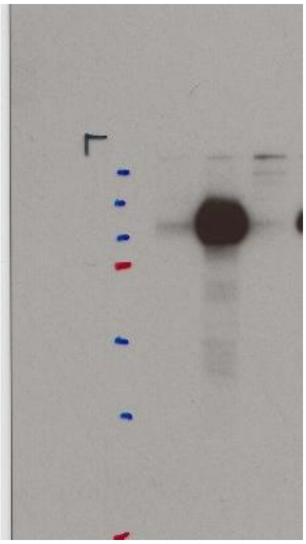

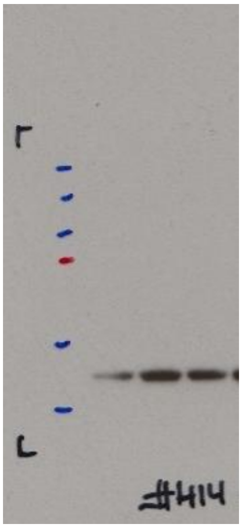

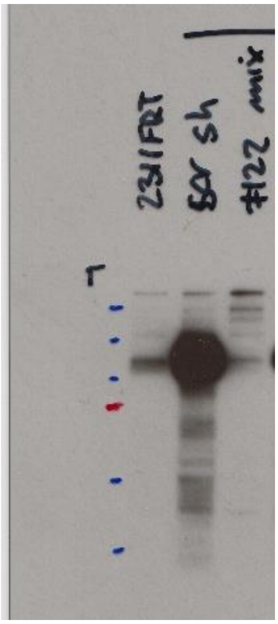

larger areas of the
blots used in Fig 2b

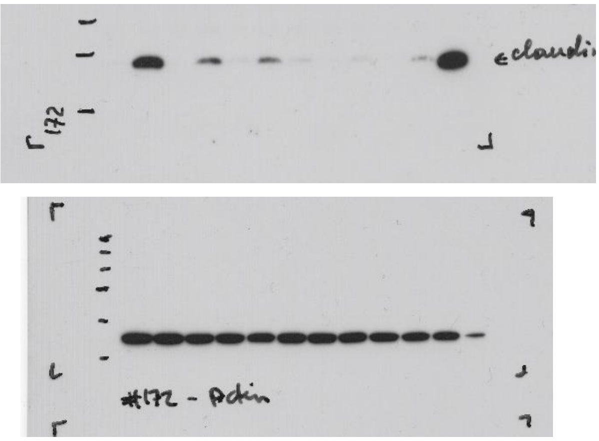

larger areas of the blots used in Fig 4a

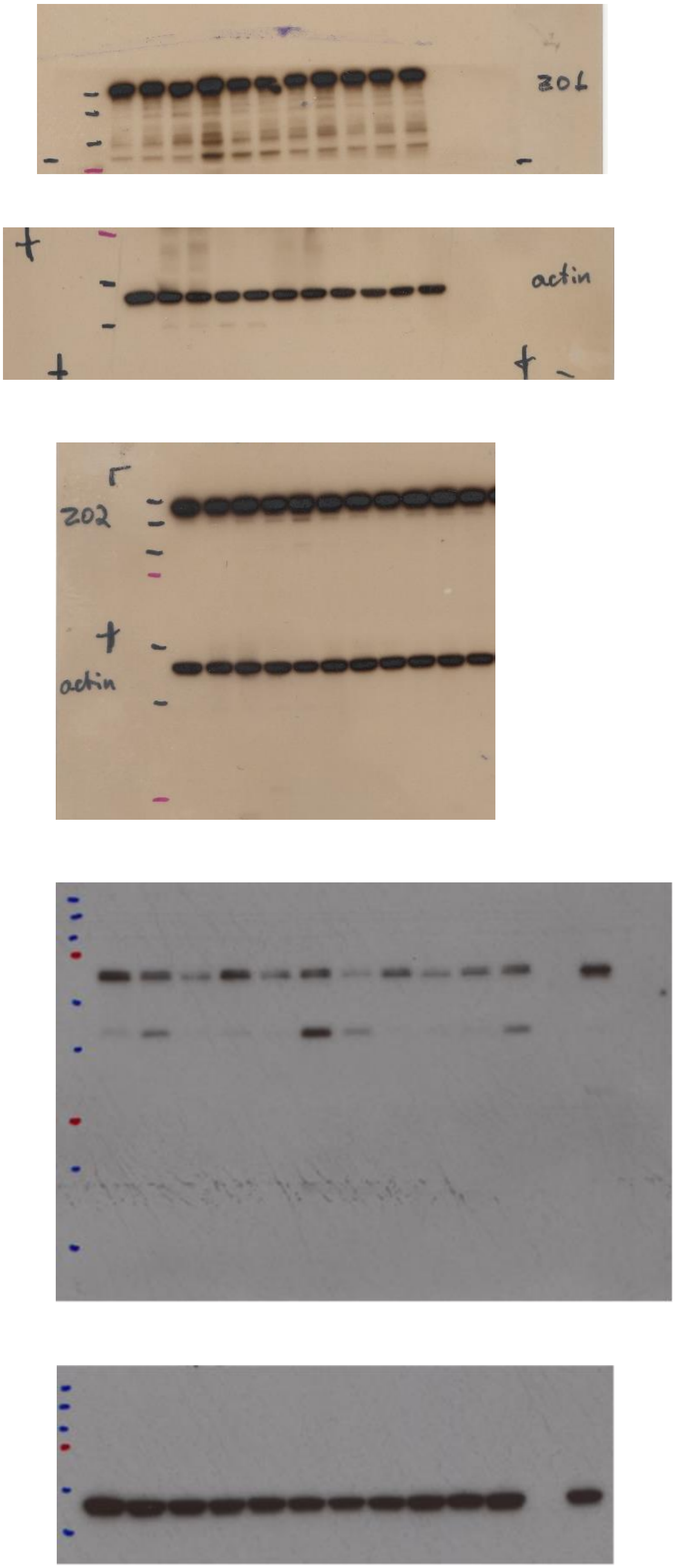

larger areas of the blots used in Fig 4c

MDA NT #22 #22 #23 #51 #52 #52 #71

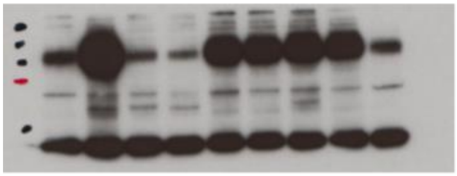

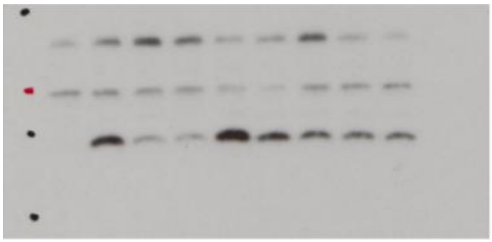

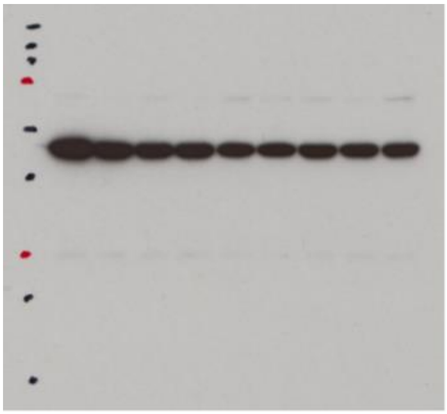

T47D NT 2 5 7 10 10 14

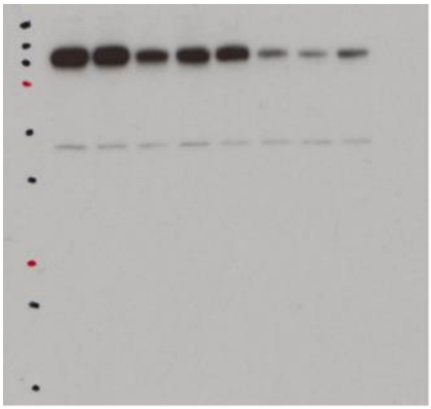

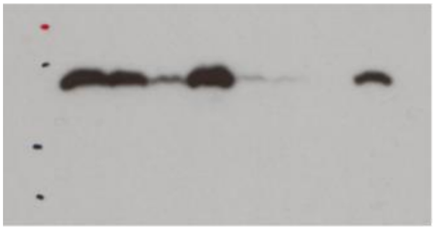

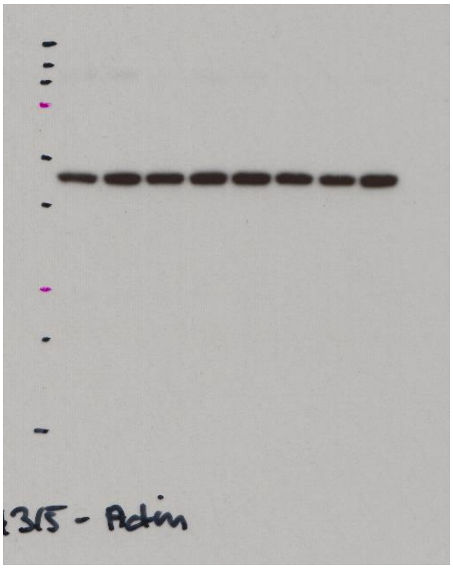

larger areas of the blots used in Fig 4d

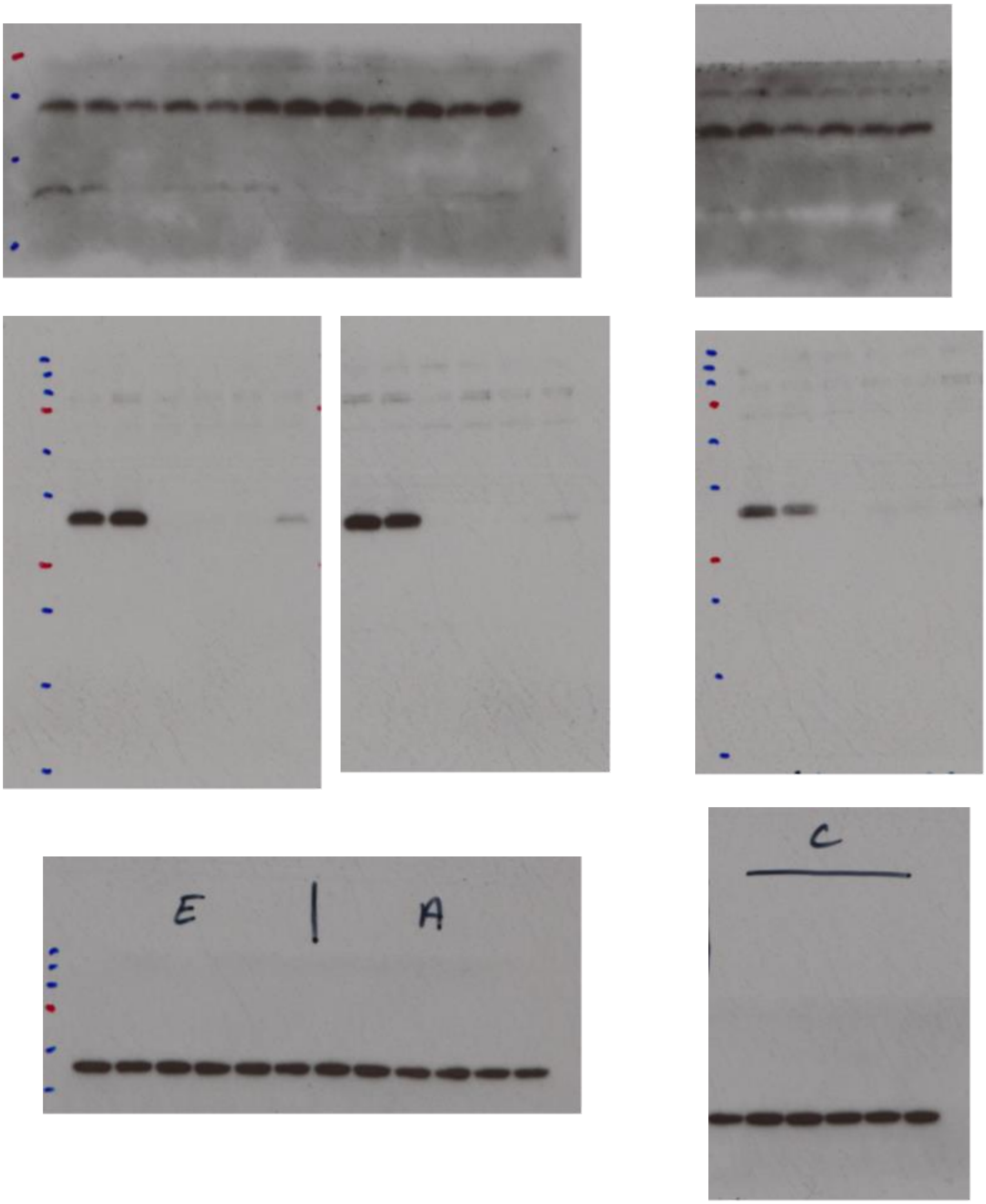

larger areas of the blots used in Fig 5a

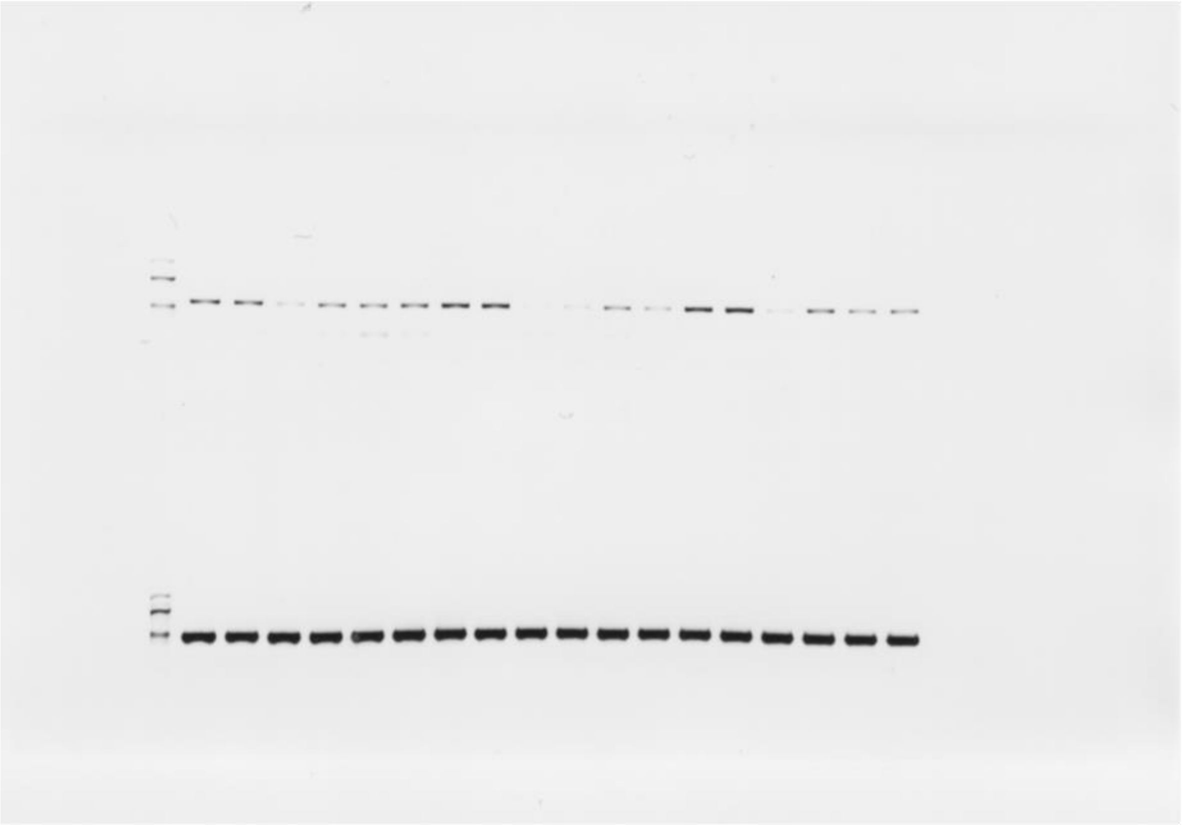

Agarose gel photo with the RT-PCR results shown in Fig 5b

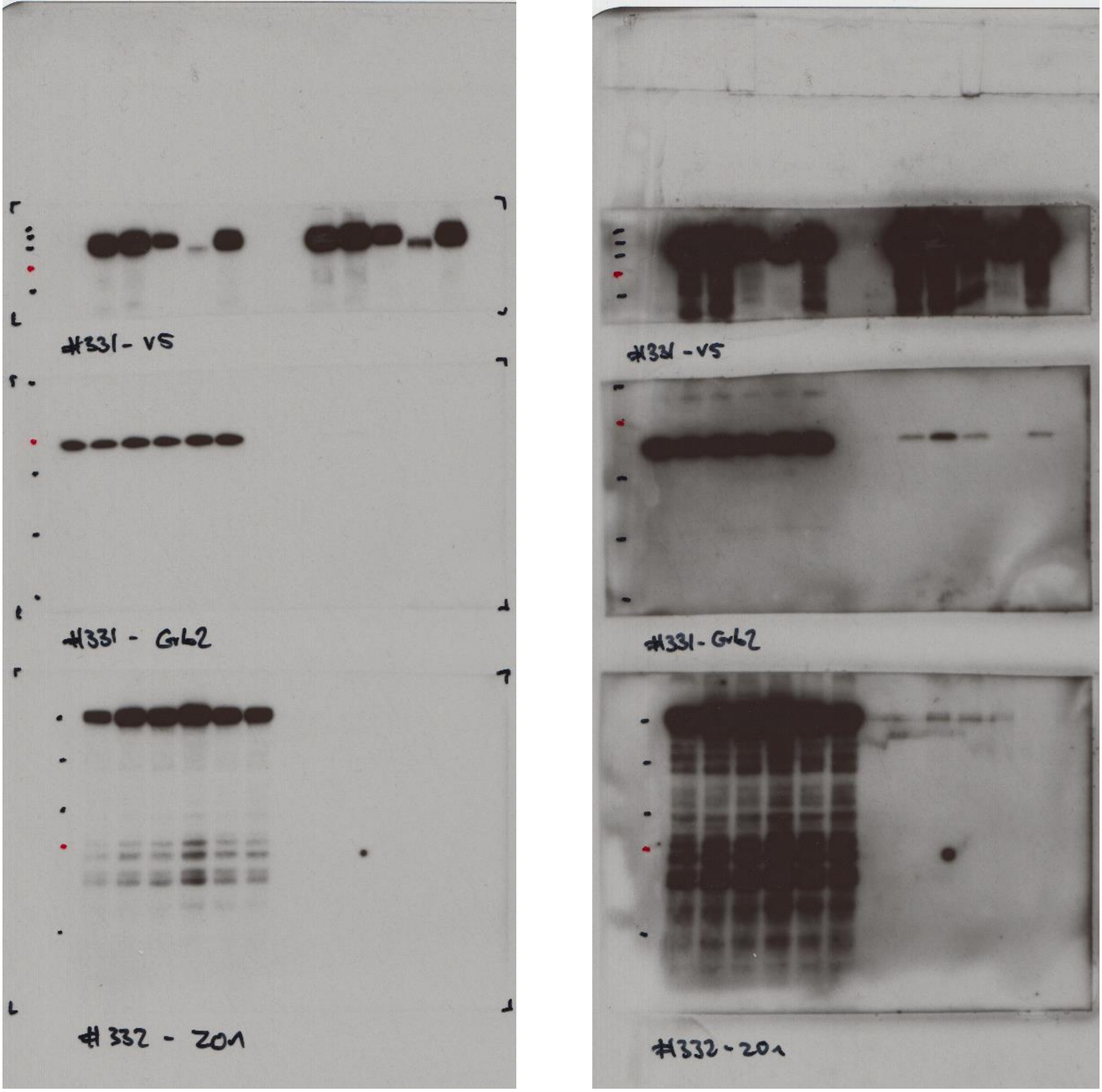

larger areas of the blots used in Fig 6e.

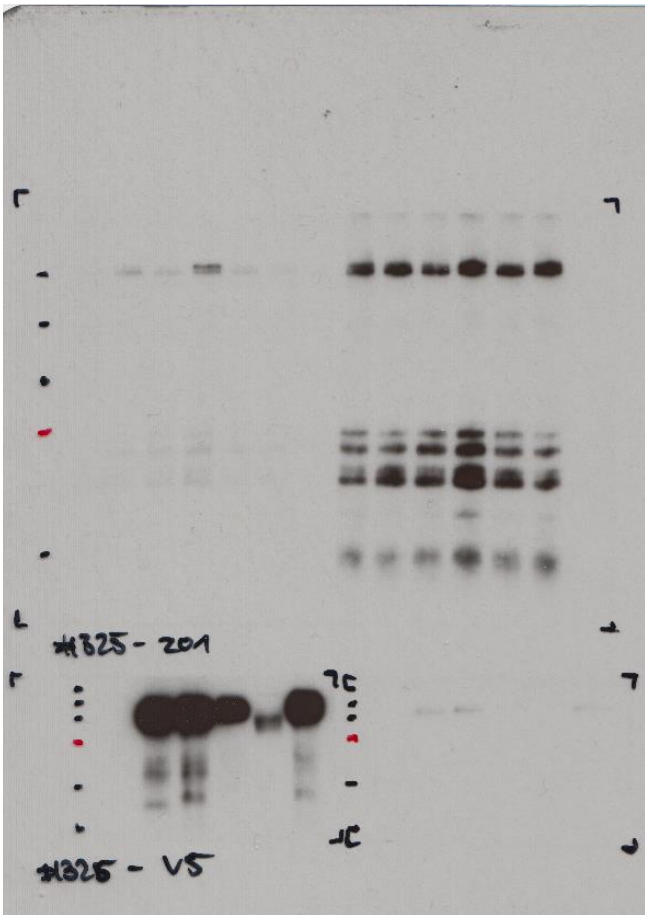

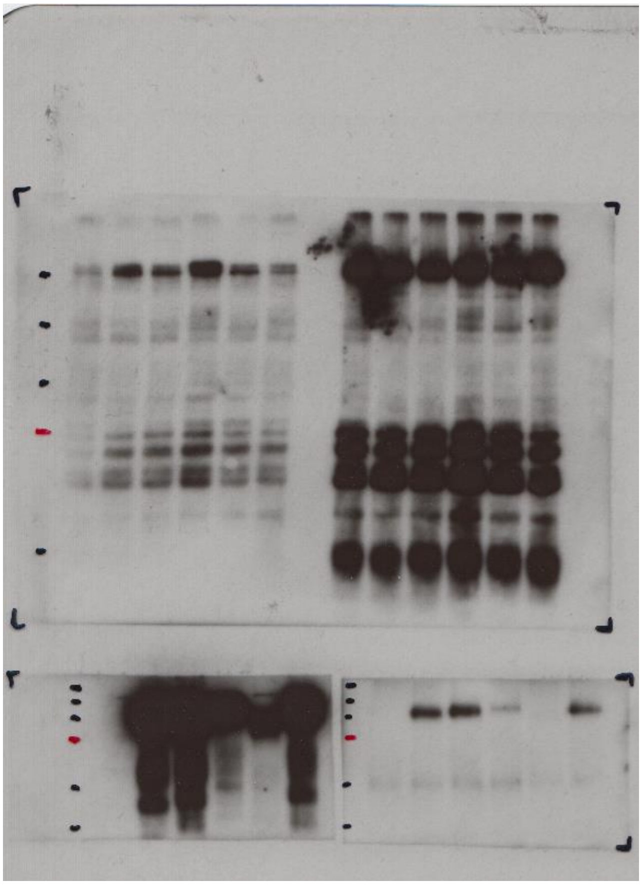

larger areas of the blots used in Fig 6f.

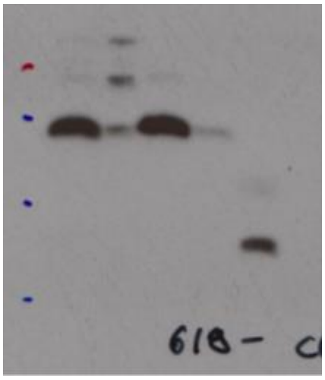

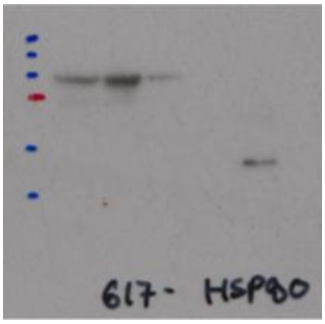

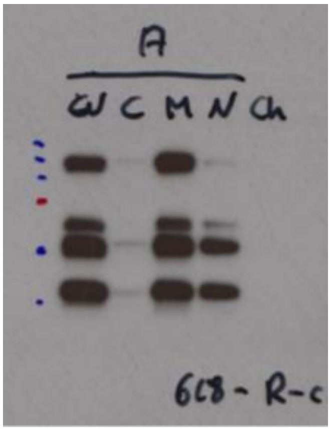

larger areas of the
blots used in Fig 6h.

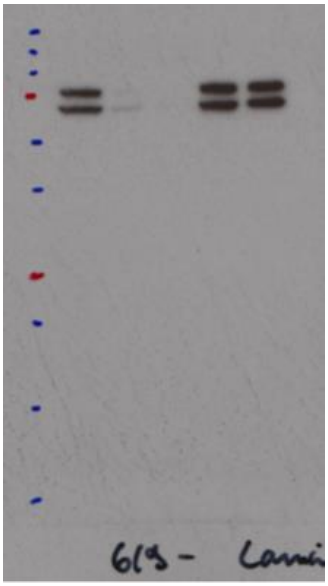

Supplement: Supplementary file 1 — Supplementary information [file 41598_2019_49021_MOESM1_ESM.pdf]
